# Shedding Light on Thermally Induced Optocapacitance at the Organic Biointerface

**DOI:** 10.1021/acs.jpcb.1c06054

**Published:** 2021-09-15

**Authors:** Gaia Bondelli, Samim Sardar, Greta Chiaravalli, Vito Vurro, Giuseppe Maria Paternò, Guglielmo Lanzani, Cosimo D’Andrea

**Affiliations:** † Department of Physics, Politecnico di Milano, 20133 Milan, Italy; ‡ Center for Nano Science and Technology @PoliMi, Istituto Italiano di Tecnologia, 20133 Milan, Italy

## Abstract

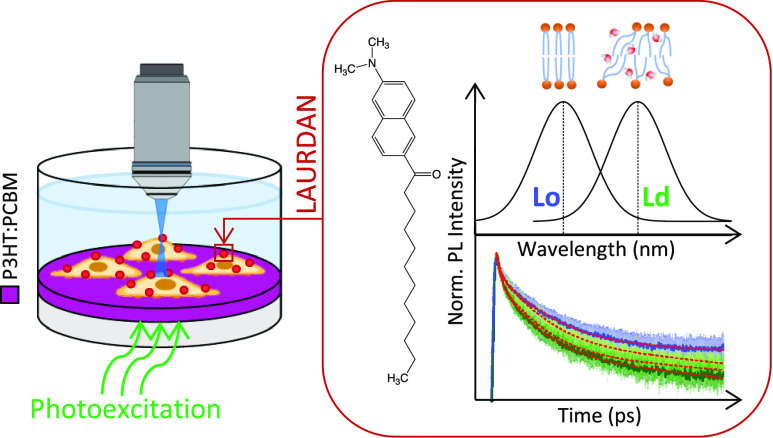

Photothermal perturbation of the cell membrane is typically achieved using transducers that convert light into thermal energy, eventually heating the cell membrane. In turn, this leads to the modulation of the membrane electrical capacitance that is assigned to a geometrical modification of the membrane structure. However, the nature of such a change is not understood. In this work, we employ an all-optical spectroscopic approach, based on the use of fluorescent probes, to monitor the membrane polarity, viscosity, and order directly in living cells under thermal excitation transduced by a photoexcited polymer film. We report two major results. First, we show that rising temperature does not just change the geometry of the membrane but indeed it affects the membrane dielectric characteristics by water penetration. Second, we find an additional effect, which is peculiar for the photoexcited semiconducting polymer film, that contributes to the system perturbation and that we tentatively assigned to the photoinduced polarization of the polymer interface.

## Introduction

The possibility to control selectively the electrical activity of living cells with low invasiveness has opened up new therapeutic paths in neurodegenerative medicine.^1^ Optical technologies are particularly suited for this purpose, as light enables precise and localized perturbation of cell activity in a remote and spatiotemporal precise manner. The cell membrane is the natural target for this approach.^2−5^ In particular, membrane potential modulation occurs via the photoinduced modification of the cell membrane electrical properties, such as resistance, capacitance, and resting potential,^2^ either through direct photostimulation^6^ or using selected transducers that are able to convert light into an electrical, mechanical, chemical, or thermal stimulus.^7−11^ In the last decade, organic semiconductors have emerged as powerful photoactuators for the development of functional interfaces with living cells and organisms. These systems, which have benefited from a long period of development and refinement for applications in organic photovoltaics,^12^ exhibit a relatively high absorption coefficient (α ∼ 10^5^ cm^–1^) in the visible spectral range, which is suitable for light biostimulation. In addition to this functional quality, another important advantage is represented by the fact that organic semiconductors are essentially biomimetic compounds, being mostly composed of hydrogen and carbon atoms as it happens for biomolecules. This allows establishing a virtually seamless interface with biomolecules where both ionic and electronic processes can play a role.^13^ Taken together, these features make them ideal candidates for noninvasive optostimulation, for both in vitro and in vivo applications.^9,10,14−22^ Driven by these strong motivations, many efforts have been dedicated to shed light on the biophysics underpinning photostimulation with organic semiconductors.^2,16,23−25^

Here, we focus our attention onto a regime of abiotic/biotic interaction that takes place for relatively high light intensity of excitation (>1 mW/mm^2^) and long interaction time (seconds). Under such conditions, the local rise in temperature is the main phenomenon driving membrane potential dynamics. Direct photothermal stimulation was first demonstrated using infrared light that heats up water,^6^ an effect that leads to a rapid local temperature rise and a consequent increase of membrane capacitance, which is associated with a transient depolarization. However, this method can be rather spatially imprecise because of the nonlocalized water absorption process and, in addition, can lead to long-term photodegradation effects. For these reasons, various light-to-heat transducers able to confine spatially the thermal effect have been recently proposed. For instance, gold quantum dots have been employed for these purposes, owing to their large plasmonic oscillator strength and their biocompatibility.^26^ In organic semiconductors, which represent the subject of our investigations, the heating process originates from the nonradiative recombination of the photogenerated states. If we consider a planar abiotic/biotic interface, in which living cells are cultured on top of a conjugated polymer film, the local temperature at the interface can be increased of several degrees by stimulation in the absorption region of the polymer and with light densities >1 mW/mm^2^. Within this context, Martino et al. observed a photoinduced increase in membrane capacitance after excitation of poly(3-hexylthiophene) (P3HT) alone and in blend with the electron-acceptor [6,6]-phenyl-C61-butyric acid methyl ester (PCBM), which was linked to an increase of the local temperature.^16^ In addition, the authors observed that ion channel conductance was also affected, leading to a two-phase response consisting of a depolarization followed by a hyperpolarization.^16^

Regardless of the origin of the temperature increase, the corresponding increment in cell membrane capacitance emerges as a universal behavior, with a rate of ∼0.3%/°C. According to the simple plane capacitor model, a geometrical interpretation of the phenomenon based on thermal expansion would lead to an opposite outcome, namely, a capacitance decrease. Shoham et al.^27^ reconceived the model used by Shapiro^6^ introducing the ad-hoc conjecture of a thermally induced phase transition^28^ leading to the increase in capacitance. Indeed, under physiological conditions cell membranes are close to the phase transition temperature, and a slight temperature increase might lead to a structural change from gel to liquid phase of the cellular membrane bilayer.^29^ For example, the tilting of the lipid tails would reduce the membrane thickness and increase the membrane area, supporting the geometrical interpretation.^30^ The change in morphology may however be of different nature, that is, associated with the modification of the membrane composition, while the variation of membrane capacitance can be related to a change of the dielectric constant. Hence, the exact stimulation mechanism is still a matter of debate.

In this work, we employ an all-optical spectroscopic approach to shed light on these aspects. By probing the membrane polarity, viscosity, and order in living cells via well-established fluorescent probes, we provide solid evidence for the temperature-induced structural modification occurring in the membrane. Additionally, our results suggest that water permeates more favorably the disordered phase, and thus the resulting change in the dielectric response of the plasma membrane might well be the ruling phenomenon explaining the temperature-induced enhancement in capacitance. Furthermore, we find experimental evidence that optostimulation mediated by a photovoltaic organic film brings about an additional phenomenon reinforcing the perturbation effect. We tentatively assigned this to the surface charging at the biointerface. Our results suggest that optostimulation mediated by organic semiconductors is not simply due to thermal effects, but it is also related to the capability of the photogenerated charges to polarize the plasma membrane.

## Methods

## Substrate Preparation

Regioregular P3HT (99.995% purity, *M*_n_ ≈ 54,000–75,000) was purchased from Sigma-Aldrich; PCBM [C70] (99.5% purity) was purchased from Nano-C; they were both used without any further purification. The substrates for cell cultures consisted of round glass coverslips (diameter 18 mm, VWR) and carefully rinsed in a successive ultrasonic bath of nanopure water, acetone, and isopropanol. A P3HT:PCBM (1:1 wt) solution was prepared in chlorobenzene at a final concentration of 10 mg/mL. It was spin-coated on the cleaned substrates with a two-step procedure: (i) 3 s at 800 rpm and (ii) 60 s at 1600 rpm, resulting in a final film thickness of around 100 nm. For specific control measurements, the coloring pigment CI 15850 (D&C Red No.7) was dissolved in *n*-butyl acetate, and its deposition process was carefully controlled to obtain films with comparable optical density at the selected excitation wavelength (λ = 561 nm, see Figure S1). All films were thermally treated in an oven at 120 °C for 2 h for annealing and sterilization. To promote adhesion, samples for cell cultures were coated with fibronectin (from bovine plasma, Sigma-Aldrich) at a concentration of 2 μg/mL in phosphate buffered saline (PBS) for at least 30 min at 37 °C and then rinsed with PBS.

### Cell Culture Maintenance

Both confocal imaging and time-resolved photoluminescence (TRPL) in vitro measurements were performed using the immortalized cell line HEK-293 (human embryonic kidney), purchased from ATCC. HEK-293 cells were cultured in T-75 cell culture flasks containing Dulbecco’s modified Eagle’s medium–high glucose (DMEM-HG), supplemented with 10% heat-inactivated fetal bovine serum (Gibco, UK), 2% penicillin/streptomycin, and 1% l-glutamine. Phenol red-free medium was preferred, to reduce background fluorescence in subsequent experiments. Culture flasks were maintained in a humidified incubator at 37 °C with 5% CO_2_. When at confluence, cells were enzymatically dispersed with a 1x trypsin–EDTA solution, plated on the substrates, and left to grow.

### TRPL Setup

In the setup used for TRPL measurements, the light source was provided by a Ti:Sapphire oscillator (Chameleon Ultra II, Coherent) producing pulses of 140 fs with a repetition rate of 80 MHz. Second harmonic generation was obtained using a barium borate crystal; two absorbing BG40 filters removed residuals of the fundamental. Spatial resolution was achieved through a microscope incorporated in the setup. The excitation beam (370 nm) was reflected off a suitably chosen dichroic mirror (LP435) before being coupled into the objective and focused onto the sample, obtaining an excitation spot diameter of 4–5 μm and an average power of 25 μW. To achieve high excitation efficiency, a 63x water immersion objective (Leica HCX APO L 63X) with a high numerical aperture (NA 0.9) was required. Sample emission was collected by the same objective and transmitted through the dichroic mirror, and furthermore, an LP395 absorbing filter was used to remove the residual pump scatter. The microscope field of view was selected by a flip mirror and a CMOS camera (ORCA-Flash 2.8, Hamamatsu), allowing accurate positioning of the sample relative to the excitation beam via a sample XYZ differential micrometer translation stage. The emission signal was focused on the entrance slit of a spectrograph (Acton SP2300i, Princeton Instrument). The dispersed image was then focused on the entrance slit of a streak camera (Hamamatsu C5680), equipped with the Synchroscan sweep module. For Laurdan measurements, the maximum voltage applied to the sweep electrodes was such as the temporal window acquired had a time width of 2 ns, with an instrument response function of ∼20 ps. A CCD (Hamamatsu ORCA-R2 C10600) recorded the streak image. For the simultaneous excitation of the P3HT:PCBM film, a 561 nm continuous wave (CW) DPSS laser (SLIM-561-150, Oxxius) was employed, whose beam was collimated to a final diameter of 1 mm, striking the sample from the bottom (see Figure 3c). Its power density was adjusted by placing an optical attenuator in the excitation path. To avoid photoluminescence (PL) contribution from the CW laser and the polymeric film, notch 561 and FES550 filters were used in the collection path.

### Confocal Imaging and Spectroscopic Measurements with Laurdan

Laurdan (6-lauryl-2-dimethylamino-napthalene) was purchased from Sigma-Aldrich, UK. For confocal imaging and TRPL measurements, a 5 mM stock solution of Laurdan in dimethyl sulfoxide (DMSO, Sigma-Aldrich, UK) was prepared. Laurdan staining of live HEK-293 cells was performed removing the medium from the cell culture dish, prepared as described in the cell culture section above, and replacing it with 1 mL of fresh, serum-free DMEM, as the presence of serum increases background fluorescence and sequesters the dye during incubation. Laurdan was then added at a final concentration of 10 μM, known to efficiently stain cells without affecting their viability.^31^ Cells were incubated at 37 °C under a humidified 5% CO_2_ atmosphere for 30 min in the dark. For confocal microscopy measurements, Laurdan was incubated for much longer, to have an overview of the full cellular membranous environment, including the intracellular membranes. After incubation, cells were washed with warmed PBS to remove cell-unbound molecules and ready for measurements.

For confocal imaging of Laurdan-stained cells, samples were mounted on an inverted confocal laser-scanning microscope (Nikon Eclipse T*i*2, Nikon Instruments), and acquisitions were performed using an Olympus 60× oil objective. Cells were excited with a 403 nm diode laser, and the fluorescence was collected in two detection channels: 425–475 nm and 500–550 nm. Confocal images were analyzed with Fiji (ImageJ), and the generalized polarization (GP) fluidity map was realized through MATLAB.

For TRPL measurements, samples were placed on the stage of the TRPL microscope, and Laurdan PL was obtained under excitation at 370 nm. Each measurement lasted no more than 2 min, to reduce cellular stress during excitation. Because of high intrinsic cell membrane heterogeneity, the recorded spectra and dynamics slightly differ from cell to cell and from different spots of the same cell. For this reason, the cell area of interest was fixed while performing consecutive measurements at increasing temperatures, to avoid influencing the Laurdan response by changing the cell spot under investigation. The shape and general aspect of each sampled cell were checked after each measurement through the wide-field CMOS camera, to ensure that the cell did not undergo any change in shape or position. For each sample, three–four measurements were performed, and the samples were changed after about 40 min from the end of Laurdan incubation, to avoid excessive internalization. TRPL measurements at different temperatures were carried out placing the sample chamber on top of a commercial Peltier plate, whose voltage was adjusted via a source meter (Agilent, B2912A). Emission spectra were fitted with two Gaussians accounting for the Lo and Ld phases, whose center (*x*_c_) was fixed to 460 and 510 nm, respectively. For the GP analysis, formula 5 was adjusted to the following one:
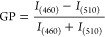
1where *I* corresponds to the area underneath the Gaussian curve in consideration. The statistic was made on about six different cells per each sample condition.

### Anisotropy Fluorescence Measurements with TMA-DPH

1-(4-trimethylamino)phenyl)-6-phenylhexa-1,3,5-triene (TMA-DPH) was purchased from Invitrogen. Introduced by Prendergast et al.^32^ in 1981 as a model molecular rotor for phospholipidic membranes, TMA-DPH interacts with living cells by instantaneous partition between the plasma membrane and the external medium. With fluorescence anisotropy, membrane fluidity is interpreted in terms of hindrance to rotational motion of the probe, because it is embedded in the constraining phospholipidic membrane array.^33^ To measure the decay of fluorescence anisotropy with TMA-DPH, polarizers were placed in the excitation and emission path. Decays of vertically, *I*(*t*)_vv_, and horizontally, *I*(*t*)_vh_, polarized fluorescence intensities with respect to the excitation beam were measured. Time-dependent anisotropy was calculated as:
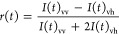
2where *I*(*t*)_vv_ and *I*(*t*)_vh_ represent the components of the light intensity emitted, respectively, parallel and perpendicular to the direction of the vertically polarized excitation light. For these fluidity measurements, HEK-293 cells were incubated with TMA-DPH for a short time (10 s) at room temperature, to label the plasma membrane. The final concentration of the probe in PBS was 2 μM, starting from a 5 mM stock solution in dimethylformamide.

### Photovoltage (PV) Measurements

Electrochemical measurements have been conducted using a potentiostat and an electrochemical cell in a three-electrode setup. The potentiostat used was a Metrohm PGSTAT302N. The reference electrode was saturated Ag/AgCl (Metrohm 60733100), and the counter electrode was a Pt wire (Metrohm 60301100). Samples were connected as the working electrode using crocodile clips in direct contact with ITO over a portion of the sample not covered by the P3HT:PCBM film. The used electrolytic solution was PBS. The illumination was supplied by a green light-emitting diode (LED) (Thorlabs M530L3-C5, 530 nm), driven by a DC2200 LED driver. The LED was placed at a distance of 25 cm from the sample and operated at maximum power. The intensity of light hitting the sample was 500 W/m^2^. PV recordings were acquired operating the potentiostat in high-speed mode using a sample rate of 10^4^ s^–1^. PV curves were all zeroed to the open-circuit potential value.

### Zeta Surface Potential Measurements

Zeta surface potential measurements have been conducted with a Zetasizer Nano with the surface zeta potential cell with a height adjustable sample holder. The cell was submerged in a solution containing silica nanoparticles, used as tracers. Measurements were performed at 125, 250, 375, 500, and 625 μm from the P3HT:PCBM sample surface. A final measurement at 1000 μm was also performed to estimate the tracer potential only because of the electrophoretic effect. The surface zeta potential was then computed as:
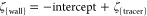
3where ζ_{wall}_ is the surface potential at the interface between P3HT:PCBM and electrolyte, intercept is the linear regression of the distance-dependent data up to the zero displacement point, and ζ_{tracer}_ is the potential of the silica nanoparticles without any contribution from the potential of the surface.

### Drift-Diffusion Simulations

The theoretical work was based on the use of a time-dependent version of the drift-diffusion model to describe the transport mechanisms of P3HT:PCBM in 1D. The mathematical model consists of two continuity equations accounting for the drift and diffusion of photogenerated holes and electrons and a Poisson equation for the electric field and potential. The computational algorithm was coded in-house and has been implemented using the MATLAB scientific environment. The generation of charge carriers is described with the use of the Lambert Beer law, while the recombination process is described with the Langevin model. At *x* = 0, the evaluation of the PV is performed, defined as PV = ψ(*x* = 0), where ψ is the electric potential. The boundary condition at the interface between P3HT:PCBM and the electrolyte is described using Marcus–Gerischer theory:
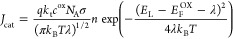
4where *q is* the elementary charge [C], *k*_t_ is the tunneling constant [m^4^s^–1^], *c*^ox^ is the concentration of molecular oxygen at the interface [mol m^–3^],^34^*N*_A_ is the Avogadro constant [mol^–1^], σ is the disorder parameter of P3HT [eV], *k*_B_ is the Boltzmann constant [J K^–1^], *T* is the room temperature [K], and λ is the width of the Gaussian distribution of molecular oxygen states [eV]. *E*_L_ is the energy of the LUMO and *E*_F_^OX^ is the energy corresponding to the potential of the oxygen reduction reaction. *n* is the electron number density at *x* = *L* [m^–3^]. The surface charge accumulated at the interface provides surface recombination centers for the holes of P3HT:PCBM. The time constant of this effect and *k*_t_ have been estimated from the fitting with the experimental data. See the Supporting Information for more details.

## Results

### Optical Probing of Membrane Fluidity and Order

**Figure 1:**
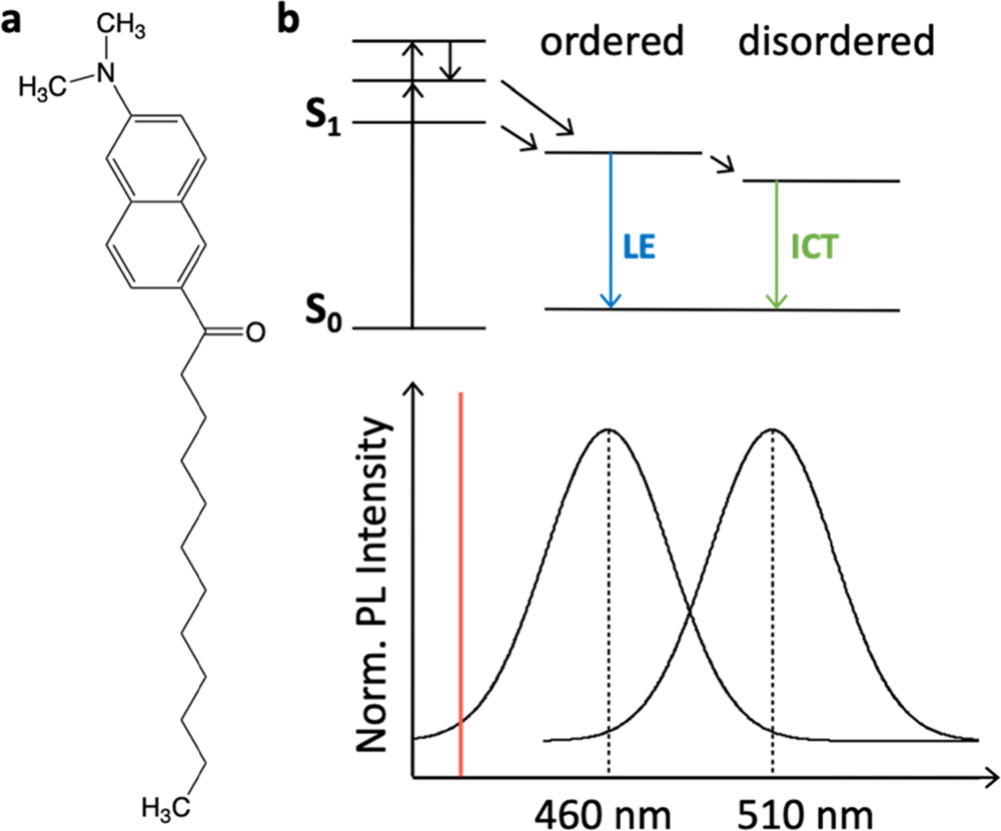
Laurdan as a polarity-sensitive probe. (a) Laurdan chemical structure. (b) Laurdan Jablonski diagram, illustrating absorption (exc. 370 nm, red line), solvent relaxation to, and emission from two different excited states: LE state, with emission near 460 nm, and ICT state in more polar environments, with emission at longer λ. A schematic representation of the corresponding emission maxima of Laurdan in ordered and disordered phases is shown.

Biological membranes are complex systems composed of a wide variety of lipids, sterols, and proteins,^35,36^ that can exist in a variety of phases, from liquid-ordered (Lo) to liquid-disordered (Ld). Different phases coexist in the membrane, and interchanging between them occurs at physiologically relevant temperatures.^37−40^ A broad term commonly adopted to describe the physical state of biological membranes is fluidity, referring to the overall Lo/Ld membrane phase ratio. The maintenance of physiological cell membrane fluidity is a prerequisite for proper membrane function, as it is associated with cell viability and normal cell growth and division.^41^ In addition, this parameter influences the dynamics of membrane proteins,^42^ which in turn affects the likelihood of protein–protein interactions and hence the efficiency of signaling pathways. Fluidity can be associated with the degree of lipid packing, the thickness of the lipid bilayer, and the rotational freedom of lipids.^31^*The degree of lipid packing can be assessed from the polarity of the bilayer*. A more compact lipid arrangement excludes polar water molecules from the otherwise nonpolar bilayer, resulting in a lowering of local environmental polarity, which can ultimately be evaluated by employing polarity-sensitive fluorescent probes. In this regard, one of the most popular and established fluorescence probes is Laurdan (Figure 1a).^31,43^ Laurdan is an amphiphilic polarity-sensitive dye, intrinsically nonfluorescent in water. When intercalates into the membrane bilayer, the molecule is however fluorescent with at least two excited states (Figure 1b): an intrinsic locally excited (LE) state and an internal charge transfer (ICT) state characterized by a large permanent dipole moment. The latter is stabilized by the reorientation of the surrounding water molecules with the Laurdan dipole moment,^44^ so that the frequency of the emitted photons is decreased.^44^ Thus, the emission spectrum of the dye undergoes a shift to longer wavelengths in more polar environments.^45−49^ This property, commonly known as solvent relaxation,^50^ makes such solvatochromic dye perfect for probing lipid phases, as the more loosely packed Ld phase presents much higher hydration than the tightly packed Lo phase, resulting in a significant fluorescence spectrum shift. Moreover, Laurdan partitions equally between Lo and Ld, giving comparable quantum yields in both phases.^51,52^ The emission lineshape allows a quantitative assessment of the membrane order that is traditionally expressed by the GP^31,53^ parameter, which is defined as:
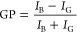
5where *I*_B_ and *I*_G_ correspond to the intensities at the blue and green edges of the emission spectrum, respectively, for a given excitation wavelength.^54^ Because the GP value is a ratio of two intensities, measurements do not depend on local probe concentrations or surface area. Importantly, the membrane-phase-dependent emission shifts have been reported to be independent of the nature of the glycerophospholipid polar head group.^55^

Even though steady-state GP measurements can provide useful estimates of the overall lipid environment,^56^ an analysis of the fluorescence lifetime of Laurdan can provide further information, as the local membrane environment affects the excited state dynamics. Laurdan excited states are in fact sensitive to collisional quenching by water molecules within the membrane.^57−59^ Consequently, a decrease in Laurdan fluorescence lifetime can be assigned to an increase in water content of the membrane^45,59^ and, thus, to a more disordered phase.

We analyzed the fluorescence properties of Laurdan internalized in the membrane of live HEK-293 cells, grown on two different substrates: a calibration sample where the substrate is glass and the temperature increase is provided by the use of a Peltier plate; and a spin-casted film of P3HT:PCBM (1:1 wt) blend, which was photoexcited with a CW laser at 561 nm. TRPL measurements with picosecond temporal resolution were carried out to record the entire time course of the Laurdan spectral shift immediately after excitation.

### Confocal Imaging and TRPL Measurements on HEK-293 Cells Plated on Glass Samples

**Figure 2:**
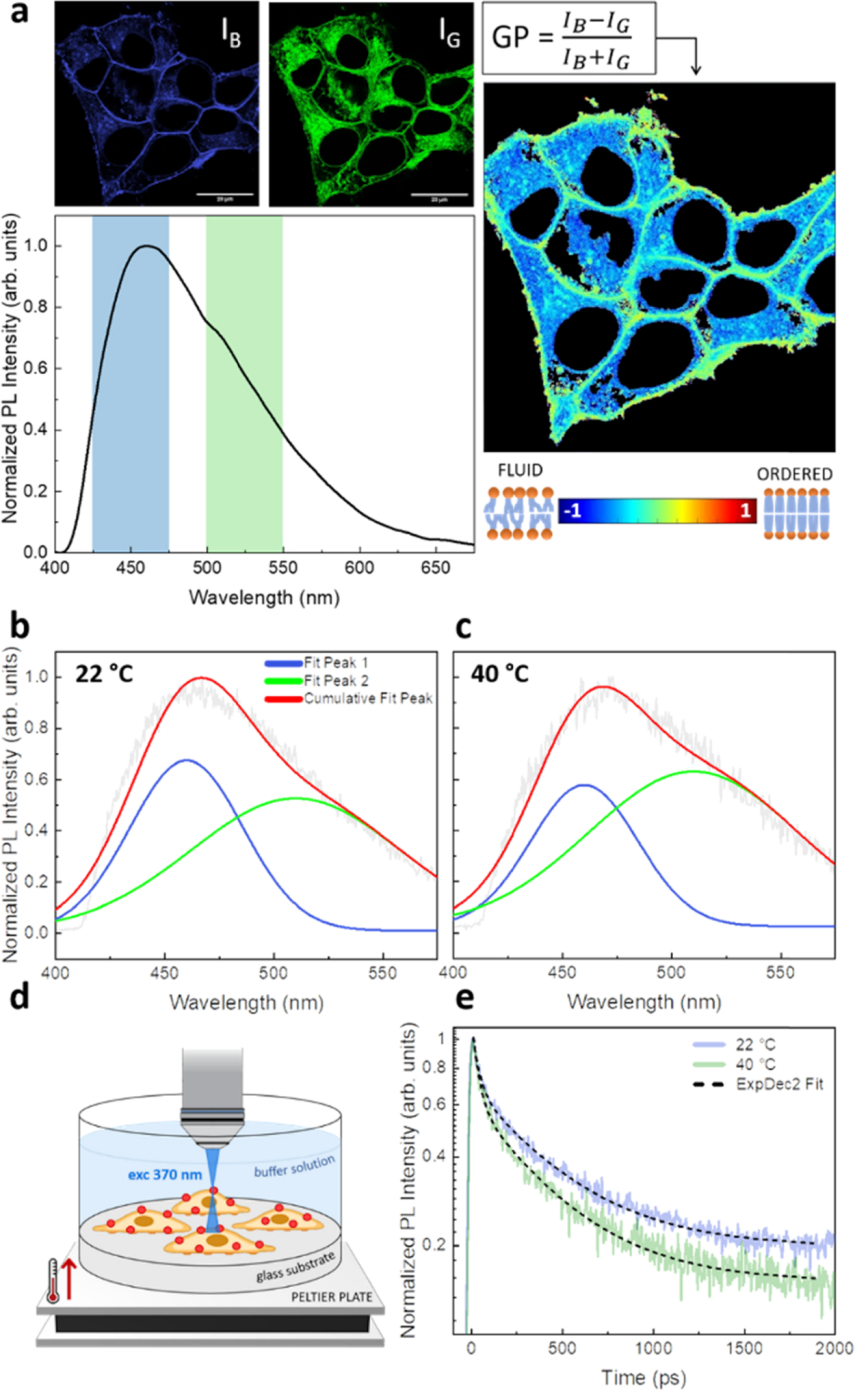
Laurdan probe monitors membrane fluidity in cells through a shift in the emission spectrum. (a) Laurdan fluorescence images of HEK-293 cells recorded simultaneously in the blue channel (425–475 nm, *I*_B_) and green channel (500–550 nm, *I*_G_) and relative Laurdan emission spectrum. On the right, fluidity map obtained applying the GP formula for each pixel, pseudocolored as indicated by the scale with GP ranging from −1 to 1. The scale bar is 20 μm. (b–e) TRPL measurements on HEK-293 cells grown on glass substrates. Deconvolution of the Laurdan emission spectra in HEK-293 cells recorded at 22 °C (b) and 40 °C (c), upon excitation at 370 nm, fitted with two Gaussian curves centered at 460 and 510 nm, used in the GP evaluation. Obtained values go toward more negative values (disordered state). (d) Schematic representation of the sample used in this study, heated by a Peltier plate. (e) Laurdan PL kinetics, integrated in the range of 440–650 nm, fitted to biexponential decay functions: dynamics become faster when temperature is increased.

As a preliminary analysis, we stained with Laurdan live HEK-293 cells plated on glass substrates and we observed them through a confocal microscope. Figure 2a depicts Laurdan fluorescence images of HEK-293 cells excited with a 403 nm laser and recorded them simultaneously in emission ranges of 425–475 (*I*_B_) and 500–550 nm (*I*_G_). The corresponding Laurdan emission spectrum is also reported. In the HEK-293 cell membrane at room temperature (22 °C), Laurdan emission is composed of two spectral contributions centered at 460 and 510 nm, accounting for the ordered and disordered lipid phases, respectively. As discussed in the Introduction, membrane fluidity is expressed in terms of ratio of emission intensities using the GP formula, which ranges from −1 (disordered environment) to +1 (ordered environment). Here, we calculated GP for each pixel to obtain a “fluidity map” (right side of Figure 2a), where blue colors indicate a low membrane order in correspondence of intracellular membranes, whereas yellowish colors indicate a higher membrane order, matching the plasma membrane, which indeed contains the majority of membrane cholesterol, appearing thus as a “more ordered” phase. These measurements validate the use of Laurdan and provide the background for further experiments.

Calibration of the Laurdan spectral changes at different temperatures was carried out in a more versatile TRPL setup, which allowed achieving intracellular spatial resolution and hence sample specific cell spots with a diameter of around 4–5 μm (cell size ∼10 to 20 μm). The possibility to have a microscope in the upright configuration permitted to position a Peltier plate below the sample chamber (Figure 2d), whose temperature was controlled by a source meter. Upon increasing the sample temperature from 22 to 40 °C, the PL spectra of Laurdan exhibit an increase of the spectral contribution peaking at 510 nm, which is indicative of the transition to a more disordered state of the membrane. We deconvolved the experimental spectra into two Gaussian components, whose values are reported in Table S1. Figure 2b,c shows the best fit recorded at 22 and 40 °C with excitation at 370 nm. From these, we worked out the value of GP based on eq 3 to characterize the predominant phase state of the lipid domain. We found average GP starting values at 22 °C to be equal to 0.19, which are consistent with previous studies on HEK-293 cells.^60^ The resulting ΔGP = GP (40 °C) – GP (22 °C) was equal to −0.187 ± 0.02 (ΔGP/GP ≈ −1), indicating increased fluidity and membrane hydration upon increasing the temperature. Next, we measured the Laurdan PL lifetime upon changing temperature (Figure 2e). The lifetime traces of the Laurdan-labeled plasma membrane could be well fitted to double exponential decay curves^61,62^ (τ_1_ < τ_2_) plus an offset component (*y*_0_), accounting for the long component that falls outside our time-window (>2 ns). We integrated the kinetics in the range of 440–650 nm, avoiding a Raman peak associated with the aqueous buffer, centered at 430 nm. Fitted values are reported in Table 1.

**Table 1:** Laurdan Decay Curve Average Parameters (*n* = 6) Obtained at 22 and 40 °C on HEK-293 Cells Plated on Glass Substrates^a^

	22 °C	40 °C
*y*_0_	0.19 ± 0.002	0.15 ± 0.002
τ_1_	35 ± 5 ps (∼45%)	35 ± 5 ps (∼53%)
τ_2_	427 ± 8 ps (∼55%)	402 ± 9 ps (∼47%)

a Kinetics were fitted to biexponential decay functions, with an offset (*y*_0_) accounting for the long lifetime component that falls outside our time-window.

The fast component τ_1_ does not change, but it acquires a larger amplitude at higher temperature. Upon heating, τ_2_ drops by almost 6% and shows a smaller relative weight, while the offset y_0_ decreases (−21%). We evaluated the adequacy of the biexponential decay fitting by inspection of the residual distribution and by the statistical parameter reduced chi-square (χ^2^) (Figure S2). To summarize, our data indicate that cells at higher temperature (40 °C) displayed a shorter Laurdan lifetime, in agreement with the GP spectral shift. This implies an increased quenching from water molecules at the membrane–water interface and thus an increased level of water hydration, consistent with the enhanced disorder (fluidity) highlighted by steady-state measurements. This result suggests that by enhancing temperature the polarity of the membrane is increased (i.e., its dielectric constant) because of the penetration of water in the membrane. This process contributes to the increase in cell membrane capacitance that is observed by heating.

### TRPL Measurements on HEK-293 Cells Plated on P3HT:PCBM Samples

**Figure 3:**
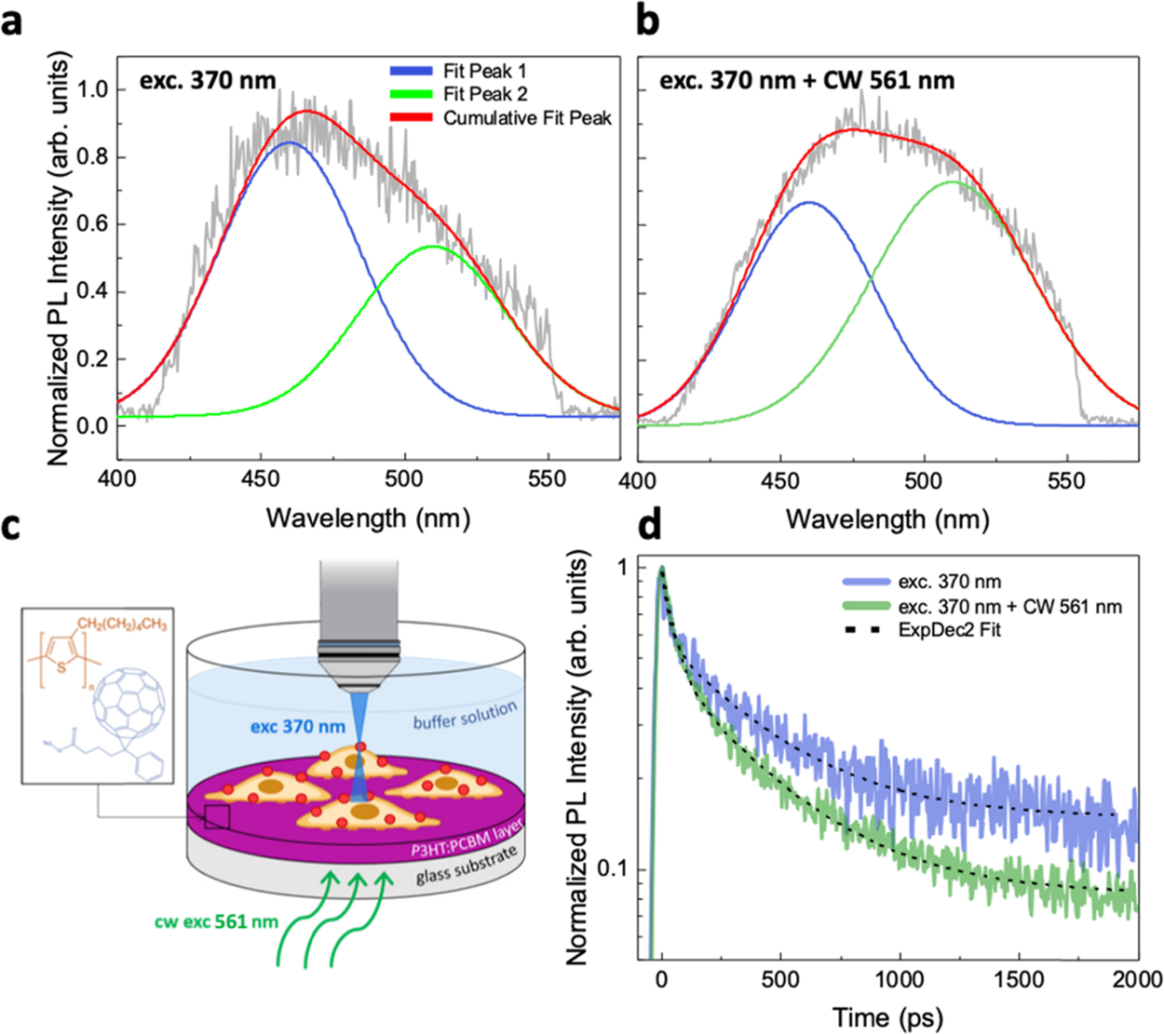
TRPL measurements on HEK-293 cells grown on P3HT:PCBM films. Deconvolution of the Laurdan emission spectra, recorded with excitation at 370 nm only (a) and at 370 nm + CW 561 nm (46 mW/mm^2^) (b), fitted with two Gaussian curves centered at 460 and 510 nm, used in the GP evaluation. Measurements were performed on the same cell spot. PL contribution from the polymeric film was avoided integrating only the range of 100–2000 ps. (c) Schematic representation of the sample used in this study. The laser beam illuminates the back of the sample, where the P3HT:PCBM film is located, while the PL excitation 370 nm beam impinges the sample from the opposite side, where the cells stained with Laurdan are plated. (d) Laurdan PL kinetics, integrated in the range of 440–550 nm, fitted to double exponential decay curves: dynamics become faster upon illumination with a 561 nm CW laser.

In the previous experiments, heating of the cell specimens was provided by the Peltier plate. Hereafter, we introduce a P3HT:PCBM thin layer spin-coated on top of the glass substrate as the photothermal agent, resulting in the same device structure employed for optical cell stimulation.^16^ Upon resonant excitation (561 nm), the polymeric film acts as a light transducer that dissipates the great majority of the absorbed photons energy via nonradiative decay and ultimately by heat transfer to the surrounding environment. Note that the PL quantum yield of the bulk heterojunction film is very small, limiting possible interference with the detection of Laurdan emission.^63^ Using finite element simulations, we estimate that the temperature variation at the interface with the polymeric film induced by illumination of the sample (*A*_light_ = 0.78 mm^2^, *I*_light_ = 46 mW/mm^2^, see the Supplementary Information) is about 16 °C at the surface, bringing the system to a final temperature of around 37 °C (Figure S3). According to the simulation, temperature reaches a stationary value in about 20 s, that is, at the beginning of the light exposure lasting 120 s. To ensure that the underlying blend film is not contributing to Laurdan PL, we performed some control measurements with the P3HT:PCBM film and aqueous buffer solution only, without the presence of cells (Figure S4). In any case, we observed a PL signal from P3HT:PCBM only in the first ∼100 ps, which we could easily discard in the subsequent analysis. We then analyzed Laurdan fluorescence properties in live HEK-293 cells grown on top of the P3HT:PCBM film, to assess directly any alterations in the membrane phase. Notice that we focus our attention onto the regime of long stimuli (seconds) and relatively high light intensity of excitation (>1 mW/mm^2^), which may be different from the one adopted in electrophysiology measurements to test membrane capacitance changes. Under the conditions employed in this work, the local rise in temperature is the main phenomenon driving membrane potential dynamics. We first performed a control measurement heating the P3HT:PCBM film through the Peltier plate from 22 to 40 °C, as previously done for the glass samples. The average values of the two Gaussian components measured are reported in Table S2. The worked out average ΔGP was equal to −0.152 ± 0.03 (ΔGP/GP ≈ −0.97), very similar to the one obtained with glass samples. This allowed us to exclude possible influences of the substrate on the cell membrane ability to react to a direct temperature change. We then carried out consecutive measurements exciting first the sample with the 370 nm pulsed laser only and then with both 370 nm and CW 561 nm lasers at the same time (lasting 120 s). As reported in Figure 3, we found that excitation of the P3HT:PCBM film leads to the enhancement of the green emission (510 nm). In this case, the average ΔGP shifted toward more negative values (ΔGP = −0.349 ± 0.04, ΔGP/GP ≈ −1.84, Figure 3a,b and Table S3), an effect which can be ascribed to a predominance of Ld lipid domains and increased membrane hydration. *We note that the measured* Δ*GPs are almost double in magnitude when compared to those measured in the Peltier plate experiment*, albeit the expected rise in temperature, for the applied power density (46 mW/mm^2^) and time duration (120 s), is slightly less than the one obtained by Peltier heating (16 vs 18 °C). We then analyzed the variation in the PL dynamics (Figure 3d). Again, we found that the results follow the same trend as those collected on heated glass samples; however, the changes obtained switching on the 561 nm CW laser are larger. We integrated the kinetics in the range of 440–550 nm and fitted to double exponential decay curves as before (see residual distribution in Figure S5). In the measured average values (Table 2), the time constant τ_2_ remarkably drops by 20%, while τ_1_ remains constant and y_0_ decreases by almost 40%.

**Table 2:** Laurdan Decay Curve Average Parameters (*n* = 6) Obtained Exciting HEK-293 Cells Plated on P3HT:PCBM Substrates^a^

	exc. 370 nm	exc. 370 nm + CW 561 nm
*y*_0_	0.15 ± 0.003	0.09 ± 0.002
τ_1_	30 ± 6 ps (∼46%)	30 ± 6 ps (∼51%)
τ_2_	388 ± 18 ps (∼54%)	310 ± 6 ps (∼49%)

a Kinetics were fitted to biexponential decay functions, with an offset (*y*_0_) accounting for the long lifetime component that falls outside our time-window.

**Figure 4:**
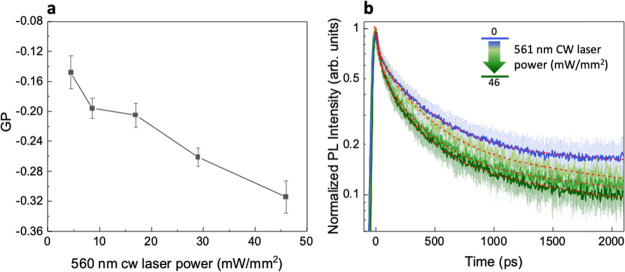
Laurdan PL as a function of 561 nm CW laser power. (a) Average (*n* = 6) ΔGP for TRPL measurement with 370 nm excitation only and TRPL measurement with 370 nm + CW 561 nm excitation; error bars represent the standard deviation. (b) Laurdan TRPL decays at increasing 561 nm CW laser power: dynamics become faster, going from 0 mW/mm^2^ (exc. 370 nm only) to 46 mW/mm^2^ and were fitted to double exponential decay curves.

We checked that the use of the CW laser without the underlying P3HT:PCBM layer does not lead to any detectable modification of the Laurdan emission (see Figure S6). Figure S7 shows that the photoinduced changes induced by P3HT:PCBM survive after switching off the laser light. Finally, we measured ΔGP using several power densities of the 561 nm light (29, 17, 8.6, and 4.5 mW/mm^2^). The results show that ΔGP is always negative and roughly linearly dependent on laser intensity (Figure 4a). In addition, Laurdan emission decay is consistently faster upon increasing intensity (Figure 4b). The results of the double exponential fitting of the decay curves are reported in Table S4. Note that the presence of the 370 nm light might lead to some heating mediated by the absorbing polymer film (a rough estimate of the upper limit is 1°). Yet what we measure is a change on top of this contribution, if any. In addition, if the 370 nm light were effective, we would detect a change in the GP when measured on top of the polymer film, while this does not happen, as the initial value is comparable to that measured on glass. Finally yet importantly, the measurement reported below on a dye film excludes this too.

We conclude that optostimulation through the organic semiconductor layer leads to bigger changes than using the heating plate, both in terms of spectral shape (ΔGP variation) and decay kinetics, albeit the induced temperature rise is comparable: this suggests the presence of an additional excitation phenomenon.

### Membrane Fluidity Assessment

As stated above, Laurdan is primarily sensitive to polarity, which in turn depends on the membrane order. To evaluate possible changes in the membrane order regardless of polarity, we probed directly the plasma membrane fluidity by measuring the fluorescence anisotropy of the membrane marker 1-(4-trimethylamino)phenyl)-6-phenylhexa-1,3,5-triene (TMA-DPH) in living HEK-293 cells. TMA-DPH fluorescence lifetime is a good indicator of the microenvironment of the fluorophore, and earlier studies have shown that TMA-DPH exhibits different lifetimes in the liquid-ordered and liquid-disordered phases.^64,65^ As shown in Figure S8a, the decay of the fluorescence anisotropy in samples at room temperature and excited at 370 nm only exhibits a monoexponential decay (τ ∼ 1.5 ns), characteristic of a gel (Lo) phase of the cell membrane. This was valid for both glass and P3HT:PCBM substrates. Heating of the samples with the Peltier plate or photoexcitation of the polymeric substrate using the CW laser leads to similar changes in the PL dynamics (τ ∼ 1.1 ns) indicating the formation of a more fluid phase in the plasma membrane following the temperature rise. Because the expected temperature rise in both situations is similar, the result is consistent with a thermally induced change of the membrane fluidity in both configurations, namely, the increase in membrane fluidity (lower order) with higher temperature.

## Discussion

Using two intramembrane fluorescent probes, we show that the cell temperature increase induces a structural relaxation of the plasma membrane toward a more fluid and less ordered phase, which favors water penetration. This offers an explanation for the observed temperature-induced capacitance enhancement, beyond the geometrical effect (smaller thickness and larger area). Water penetration in the membrane leads to higher dielectric screening, which is directly proportional to capacitance. Furthermore, we cannot exclude that water is also contributing to the reduction in electrical resistance across the membrane that was previously reported.^16^

Comparing the heating induced by a Peltier device with that induced by photoexcitation of a P3HT:PCBM layer, we find that photoexcitation is causing a larger (almost double) change in the observable linked to polarity of the cell membrane. As our direct membrane fluidity assessment with the probe TMA-DPH is consistent with a thermally induced change of the membrane fluidity in both configurations (i.e., increase in membrane fluidity with higher temperature), we conjecture the reason behind the unexpected discrepancy between photoexcitation and heating to be because of the surface electrical charging. To evaluate this scenario, the P3HT:PCBM layer was replaced by a coloring pigment CI 15850 (D&C Red No.7) film, a material that absorbs light in the same range of the organic semiconductor blend but does not support photogeneration of charges. We carefully controlled the deposition process of CI 15850 to obtain films with comparable optical density at the selected excitation wavelength (λ = 561 nm, see Figure S1a), thus able to generate a temperature increase comparable to that of P3HT:PCBM. We verified this by measuring the bath temperature variation in the close proximity of the absorbing layer using the method of the calibrated pipette resistance^66^ (Figure S1b). As reported in Figure S9, we found that upon exciting the sample with the CW 561 nm laser, the green component in the Laurdan emission spectrum (510 nm) increases only to a modest extent. Deconvolution analysis yields the average relative area values reported in Table S5, from which the calculated ΔGP is found to be −0.146 (ΔGP/GP = −0.9). This value is consistent with that obtained in the Peltier plate experiments (ΔGP = −0.18; ΔGP/GP = −1), where the only parameter in play is temperature, but it is significantly smaller than that measured for P3HT:PCBM (ΔGP = −0.349; ΔGP/GP = −1.8). The PL lifetime analysis (Figure S9c) leads to the same outcome. Kinetics fitted to double exponential decay curves display only slightly faster Laurdan lifetime when the 561 nm CW laser is switched on. In the measured values reported in Table 3, time constant τ_2_ drops by 2.4%, while τ_1_ remains constant and *y*_0_ decreases by 28%.

**Table 3:** Laurdan Decay Curve Average Parameters (*n* = 8) Obtained Exciting HEK-293 Cells Plated on CI 15850 Substrates^a^

	exc. 370 nm	exc. 370 nm + cw 561 nm
*y*_0_	0.14 ± 0.003	0.09 ± 0.001
τ_1_	50 ± 5 ps (∼53%)	50 ± 5 ps (∼57%)
τ_2_	438 ± 9 ps (∼47%)	427 ± 8 ps (∼43%)

a Kinetics were fitted to biexponential decay functions, with an offset (*y*_0_) accounting for the long lifetime component that falls outside our time-window.

We can thus conclude that photoexcitation of a “dielectric” film leads to smaller effect on the cell membrane compared to photoexcitation of a semiconductor film. A possible cause of such a discrepancy is the photogeneration of long-lived charges in the P3HT:PCBM film. To corroborate such a hypothesis, we first attempt to evaluate the charge photogeneration process in the P3HT:PCBM film interfaced with a liquid via an experimental protocol, which includes: (i) running a numerical simulation based on the drift-diffusion model; (ii) setting up a photoelectrochemical experiment to measure open-circuit PV; and (iii) measuring zeta surface potential.

The simulation reproduces the photoinduced charge carrier dynamics in a P3HT:PCBM film sandwiched between an ITO electrode and the electrolyte. The experimental observable is photovoltage as detected at the ITO electrode that accounts for the charge configuration at both interfaces. A key assumption in the model is the accumulation of photogenerated negative charges in proximity of the electrolyte because of oxygen trapping.^34,67^ The interface is far from an ideal flat plane, rather being a rough boundary defining a diffuse interface in the order of 10 nm, in which hydrated oxygen and water clusters permeate the polymer free volume. The negative charges will be distributed within the diffuse interface at the electrolyte, while the positive charges spread in the polymer bulk. The simulation provides an estimation of the accumulated charge at the interface after 120 s of illumination of σ = 8 × 10^–2^ Cm^–2^, which is coherent with the one retrieved from the experimental PV (see Figure S10). The obtained value also matches well with the surface potential measurement, providing *V* = −30 mV at about 200 μm from the surface of the sample, as visible in Figure S11. Once assessed the negative charging of the polymer surface, we discuss what this could do to our experiment on cells. The cell membrane adjacent to the polymer film surface rests on a protein layer that fills the cleft. At the close proximity of the interface, there will be a weak electric potential that we can estimate considering two disks with surface charge σ, of opposite sign, with an area equivalent to the cell one and separated by a distance δ*x*. For distances *x* from the surface such as *x* ≫ δ*x*, the electrostatic voltage is (see the Supporting Information)
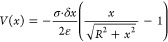
6where δ*x* is the effective separation of the negative and positive charge at the polymer electrolyte interface and *R* = 15 μm is the cell radius. The distance *x* corresponds to the cleft thickness for the adjacent side, and to the cell height for the opposite side, *x =* 0.01 μm and *x* = 10 μm, respectively. If we assume δ*x =* 1 nm, we get *V*_adj_*≈* −50 mV and *V*_opp_*= −*30 mV. The corresponding electric field is in the order of 10^3^ V/m. These numbers should not be considered as quantitative results, rather they suggest the existence of an electrical polarization that could act upon the CT emission from Laurdan, the water penetration in the membrane, the protein layer in the cleft, or the local pH.^68,69^ At this stage, we cannot speculate further.

In conclusion, we showed that water penetration in the plasma membrane is likely the major contribution to the capacitance enhancement observed in cell’s electrophysiology upon rising the temperature. Furthermore, we highlighted a peculiar effect associated with the photoexcitation of an organic semiconductor interface that may be reconciled to the steady-state charge generation following light absorption. We note here that reducing the light intensity does not allow to separate the photoinduced from the temperature-induced phenomena, as both have similar dependence. Albeit inspiring toward the understanding of the abiotic/biotic interface in general, this work explores the regime of long stimuli, in the tens of seconds, and high light intensity (>1 mW/mm^2^) well separated from the short time stimulation and low intensity adopted in electrophysiology or possibly taking place in vivo.^14^

## Supporting Information Available

Supplementary discussion on finite element simulation of thermal diffusion (Figure S3) and drift-diffusion simulations; supplementary figures of UV–vis absorption and calibrated pipette measurements of the two employed photoactive layers (Figure S1); Laurdan decay fittings for different conditions and control measurements (Figures S2, S4–S7, and S9); TMA-DPH anisotropy measurements (Figure S8); PV measurements (Figure S10); zeta surface potential measurement (Figure S11); and Supplementary tables of Laurdan GP values (Tables S1–S5) (PDF)

## References

[ref1] Potter S. M. Distributed Processing in Cultured Neuronal Networks. Prog. Brain Res. 2001, 130, 49–62. 10.1016/S0079-6123(01)30005-5. 11480288

[ref2] Manfredi G.; Lodola F.; Paternó G. M.; Vurro V.; Baldelli P.; Benfenati F.; Lanzani G. The Physics of Plasma Membrane Photostimulation. APL Mater. 2021, 9, 030901 10.1063/5.0037109.

[ref3] DiFrancesco M. L.; Lodola F.; Colombo E.; Maragliano L.; Bramini M.; Paternò G. M.; Baldelli P.; Serra M. D.; Lunelli L.; Marchioretto M.; et al. Neuronal Firing Modulation by a Membrane-Targeted Photoswitch. Nat. Nanotechnol. 2020, 15, 296–306. 10.1038/s41565-019-0632-6. 32015505

[ref4] Paternò G. M.; Colombo E.; Vurro V.; Lodola F.; Cimò S.; Sesti V.; Molotokaite E.; Bramini M.; Ganzer L.; Fazzi D.; D’Andrea C.; Benfenati F.; Bertarelli C.; Lanzani G. Membrane Environment Enables Ultrafast Isomerization of Amphiphilic Azobenzene. Adv. Sci. 2020, 7, 1903241 10.1002/advs.201903241. PMC7175258 32328424

[ref5] Paternò G. M.; Bondelli G.; Sakai V. G.; Sesti V.; Bertarelli C.; Lanzani G. The Effect of an Intramembrane Light-Actuator on the Dynamics of Phospholipids in Model Membranes and Intact Cells. Langmuir 2020, 36, 11517–11527. 10.1021/acs.langmuir.0c01846. 32903010

[ref6] Shapiro M. G.; Homma K.; Villarreal S.; Richter C. P.; Bezanilla F. Infrared Light Excites Cells by Changing Their Electrical Capacitance. Nat. Commun. 2012, 3, 736 10.1038/ncomms1742. 22415827 PMC3316879

[ref7] Bareket-Keren L.; Hanein Y. Novel Interfaces for Light Directed Neuronal Stimulation: Advances and Challenges. Int. J. Nanomed. 2014, 9, 65–83. 10.2147/IJN.S51193. PMC4024977 24872704

[ref8] Martino N.; Ghezzi D.; Benfenati F.; Lanzani G.; Antognazza M. R. Organic Semiconductors for Artificial Vision. J. Mater. Chem. B 2013, 1, 3768–3780. 10.1039/c3tb20213e. 32261129

[ref9] Benfenati V.; Martino N.; Antognazza M. R.; Pistone A.; Toffanin S.; Ferroni S.; Lanzani G.; Muccini M. Photostimulation of Whole-Cell Conductance in Primary Rat Neocortical Astrocytes Mediated by Organic Semiconducting Thin Films. Adv. Healthcare Mater. 2014, 3, 392–399. 10.1002/adhm.201300179. 23966220

[ref10] Ghezzi D.; Antognazza M. R.; MacCarone R.; Bellani S.; Lanzarini E.; Martino N.; Mete M.; Pertile G.; Bisti S.; Lanzani G.; Benfenati F. A Polymer Optoelectronic Interface Restores Light Sensitivity in Blind Rat Retinas. Nat. Photonics 2013, 7, 400–406. 10.1038/nphoton.2013.34. 27158258 PMC4855023

[ref11] Ghezzi D.; Antognazza M. R.; Dal Maschio M.; Lanzarini E.; Benfenati F.; Lanzani G. A Hybrid Bioorganic Interface for Neuronal Photoactivation. Nat. Commun. 2011, 2, 166 10.1038/ncomms1164. 21266966

[ref12] Dennler G.; Scharber M. C.; Brabec C. J. Polymer-Fullerene Bulk-Heterojunction Solar Cells. Adv. Mater. 2009, 21, 1323–1338. 10.1002/adma.200801283.

[ref13] Lanzani G. Materials for Bioelectronics: Organic Electronics Meets Biology. Nat. Mater. 2014, 13, 775–776. 10.1038/nmat4021. 24952749

[ref14] Pappas T. C.; Wickramanyake W. M. S.; Jan E.; Motamedi M.; Brodwick M.; Kotov N. A. Nanoscale Engineering of a Cellular Interface with Semiconductor Nanoparticle Films for Photoelectric Stimulation of Neurons. Nano Lett. 2007, 7, 513–519. 10.1021/nl062513v. 17298018

[ref15] Gautam V.; Rand D.; Hanein Y.; Narayan K. S. A Polymer Optoelectronic Interface Provides Visual Cues to a Blind Retina. Adv. Mater. 2014, 26, 1751–1756. 10.1002/adma.201304368. 24765649

[ref16] Martino N.; Feyen P.; Porro M.; Bossio C.; Zucchetti E.; Ghezzi D.; Benfenati F.; Lanzani G.; Antognazza M. R. Photothermal Cellular Stimulation in Functional Bio-Polymer Interfaces. Sci. Rep. 2015, 5, 8911 10.1038/srep08911. 25753132 PMC4354102

[ref17] Feyen P.; Colombo E.; Endeman D.; Nova M.; Laudato L.; Martino N.; Antognazza M. R.; Lanzani G.; Benfenati F.; Ghezzi D. Light-Evoked Hyperpolarization and Silencing of Neurons by Conjugated Polymers. Sci. Rep. 2016, 6, 22718 10.1038/srep22718. 26940513 PMC4778138

[ref18] Maya-Vetencourt J. F.; Ghezzi D.; Antognazza M. R.; Colombo E.; Mete M.; Feyen P.; Desii A.; Buschiazzo A.; Di Paolo M.; Di Marco S.; et al. A Fully Organic Retinal Prosthesis Restores Vision in a Rat Model of Degenerative Blindness. Nat. Mater. 2017, 16, 681–689. 10.1038/nmat4874. 28250420 PMC5446789

[ref19] Rand D.; Jakešová M.; Lubin G.; Vėbraitė I.; David-Pur M.; Đerek V.; Cramer T.; Sariciftci N. S.; Hanein Y.; Głowacki E. D. Direct Electrical Neurostimulation with Organic Pigment Photocapacitors. Adv. Mater. 2018, 30, 1707292 10.1002/adma.201707292. 29717514

[ref20] Jakešová M.; Silverå Ejneby M.; Đerek V.; Schmidt T.; Gryszel M.; Brask J.; Schindl R.; Simon D. T.; Berggren M.; Elinder F.; Głowacki E. D. Optoelectronic Control of Single Cells Using Organic Photocapacitors. Sci. Adv. 2019, 5, eaav5265 10.1126/sciadv.aav5265. 30972364 PMC6450690

[ref21] Maya-Vetencourt J. F.; Manfredi G.; Mete M.; Colombo E.; Bramini M.; Di Marco S.; Shmal D.; Mantero G.; Dipalo M.; Rocchi A.; et al. Subretinally Injected Semiconducting Polymer Nanoparticles Rescue Vision in a Rat Model of Retinal Dystrophy. Nat. Nanotechnol. 2020, 15, 698–708. 10.1038/s41565-020-0696-3. 32601447

[ref22] Tortiglione C.; Antognazza M. R.; Tino A.; Bossio C.; Marchesano V.; Bauduin A.; Zangoli M.; Morata S. V.; Lanzani G. Semiconducting Polymers Are Light Nanotransducers in Eyeless Animals. Sci. Adv. 2017, 3, e1601699 10.1126/sciadv.1601699. 28138549 PMC5266477

[ref23] Scanziani M.; Häusser M. Electrophysiology in the Age of Light. Nature 2009, 461, 930–939. 10.1038/nature08540. 19829373

[ref24] Vurro V.; Scaccabarozzi A. D.; Lodola F.; Storti F.; Marangi F.; Ross A. M.; Paternò G. M.; Scotognella F.; Criante L.; Caironi M.; Lanzani G. A Polymer Blend Substrate for Skeletal Muscle Cells Alignment and Photostimulation. Adv. Photonics Res. 2021, 2, 2000103 10.1002/adpr.202000103.

[ref25] Vurro V.; Bondelli G.; Sesti V.; Lodola F.; Paternò G. M.; Lanzani G.; Bertarelli C. Molecular Design of Amphiphilic Plasma Membrane-Targeted Azobenzenes for Nongenetic Optical Stimulation. Front. Mater. 2021, 7, 472 10.3389/fmats.2020.631567.

[ref26] Carvalho-de-Souza J. L.; Treger J. S.; Dang B.; Kent S. B. H.; Pepperberg D. R.; Bezanilla F. Photosensitivity of Neurons Enabled by Cell-Targeted Gold Nanoparticles. Neuron 2015, 86, 207–217. 10.1016/j.neuron.2015.02.033. 25772189 PMC4393361

[ref27] Plaksin M.; Kimmel E.; Shoham S. Correspondence: Revisiting the Theoretical Cell Membrane Thermal Capacitance Response. Nat. Commun. 2017, 8, 1431 10.1038/s41467-017-00435-5. 29127422 PMC5820289

[ref28] Plaksin M.; Shapira E.; Kimmel E.; Shoham S. Thermal Transients Excite Neurons through Universal Intramembrane Mechanoelectrical Effects. Phys. Rev. X 2018, 8, 11043 10.1103/PhysRevX.8.011043.

[ref29] Heimburg T. Thermal Biophysics of Membranes; Wiley-VCH Verlag GmbH & Co. KGaA:Weinheim, Germany, 2008.

[ref30] Heimburg T. The Capacitance and Electromechanical Coupling of Lipid Membranes Close to Transitions: The Effect of Electrostriction. Biophys. J. 2012, 103, 918–929. 10.1016/j.bpj.2012.07.010. 23009841 PMC3433620

[ref31] Owen D. M.; Rentero C.; Magenau A.; Abu-Siniyeh A.; Gaus K. Quantitative Imaging of Membrane Lipid Order in Cells and Organisms. Nat. Protoc. 2012, 7, 24–35. 10.1038/nprot.2011.419. 22157973

[ref32] Prendergast F. G.; Haugland R. P.; Callahan P. J. 1-[4-(Trimethylamino)Phenyl]-6-Phenylhexa-1,3,5-Triene: Synthesis, Fluorescence Properties, and Use as a Fluorescence Probe of Lipid Bilayers. Biochemistry 1981, 20, 7333–7338. 10.1021/bi00529a002. 7326228

[ref33] Illinger D.; Kuhry J. G. The Kinetic Aspects of Intracellular Fluorescence Labeling with TMA-DPH Support the Maturation Model for Endocytosis in L929 Cells. J. Cell Biol. 1994, 125, 783–794. 10.1083/jcb.125.4.783. 8188746 PMC2120073

[ref34] Bellani S.; Fazzi D.; Bruno P.; Giussani E.; Canesi E. V.; Lanzani G.; Antognazza M. R. Reversible P3HT/Oxygen Charge Transfer Complex Identification in Thin Films Exposed to Direct Contact with Water. J. Phys. Chem. C 2014, 118, 6291–6299. 10.1021/jp4119309.

[ref35] Singer S. J.; Nicolson G. L. The Fluid Mosaic Model of the Structure of Cell Membranes. Science 1972, 175, 720–731. 10.1126/science.175.4023.720. 4333397

[ref36] Goñi F. M. The Basic Structure and Dynamics of Cell Membranes: An Update of the Singer-Nicolson Model. Biochim. Biophys. Acta, Biomembr. 2014, 1838, 1467–1476. 10.1016/j.bbamem.2014.01.006. 24440423

[ref37] Sezgin E.; Levental I.; Mayor S.; Eggeling C. The Mystery of Membrane Organization: Composition, Regulation and Roles of Lipid Rafts. Nat. Rev. Mol. Cell Biol. 2017, 18, 361–374. 10.1038/nrm.2017.16. 28356571 PMC5500228

[ref38] Suhaj A.; Gowland D.; Bonini N.; Owen D. M.; Lorenz C. D. Laurdan and Di-4-ANEPPDHQ Influence the Properties of Lipid Membranes: A Classical Molecular Dynamics and Fluorescence Study. J. Phys. Chem. B 2020, 124, 11419–11430. 10.1021/acs.jpcb.0c09496. 33275430

[ref39] Simons K.; Ikonen E. Functional Rafts in Cell Membranes. Nature 1997, 387, 569–572. 10.1038/42408. 9177342

[ref40] Heimburg T. Physical Properties of Biological Membranes. 2009, arXiv:0902.2454.

[ref41] van Meer G.; Voelker D. R.; Feigenson G. W. Membrane Lipids: Where They Are and How They Behave. Nat. Rev. Mol. Cell Biol. 2008, 9, 112–124. 10.1038/nrm2330. 18216768 PMC2642958

[ref42] Goshima G.; Kiyomitsu T.; Yoda K.; Yanagida M. Human Centromere Chromatin Protein HMis12, Essential for Equal Segregation, Is Independent of CENP-A Loading Pathway. J. Cell Biol. 2003, 160, 25–39. 10.1083/jcb.200210005. 12515822 PMC2172742

[ref43] Weber G.; Farris F. J. Synthesis and Spectral Properties of a Hydrophobic Fluorescent Probe: 6-Propionyl-2-(Dimethylamino)Naphthalene. Biochemistry 1979, 18, 3075–3078. 10.1021/bi00581a025. 465454

[ref44] Macgregor R. B.; Weber G. Fluorophores in Polar Media: Spectral Effects of the Langevin Distribution of Electrostatic Interactions. Ann. N. Y. Acad. Sci. 1981, 366, 140–154. 10.1111/j.1749-6632.1981.tb20751.x.

[ref45] Parasassi T.; Krasnowska E. K.; Bagatolli L.; Gratton E. Laurdan and Prodan as Polarity-Sensitive Fluorescent Membrane Probes. J. Fluoresc. 1998, 8, 365–373. 10.1023/A:1020528716621.

[ref46] Viard M.; Gallay J.; Vincent M.; Meyer O.; Robert B.; Paternostre M. Laurdan Solvatochromism: Solvent Dielectric Relaxation and Intramolecular Excited-State Reaction. Biophys. J. 1997, 73, 2221–2234. 10.1016/S0006-3495(97)78253-5. 9336218 PMC1181123

[ref47] Vincent M.; de Foresta B.; Gallay J. Nanosecond Dynamics of a Mimicked Membrane-Water Interface Observed by Time-Resolved Stokes Shift of LAURDAN. Biophys. J. 2005, 88, 4337–4350. 10.1529/biophysj.104.057497. 15778437 PMC1305662

[ref48] Parasassi T.; De Stasio G.; Ravagnan G.; Rusch R. M.; Gratton E. Quantitation of Lipid Phases in Phospholipid Vesicles by the Generalized Polarization of Laurdan Fluorescence. Biophys. J. 1991, 60, 179–189. 10.1016/S0006-3495(91)82041-0. 1883937 PMC1260049

[ref49] Klymchenko A. S. Solvatochromic and Fluorogenic Dyes as Environment-Sensitive Probes: Design and Biological Applications. Acc. Chem. Res. 2017, 50, 366–375. 10.1021/acs.accounts.6b00517. 28067047

[ref50] Parasassi T.; Gratton E. Membrane Lipid Domains and Dynamics as Detected by Laurdan Fluorescence. J. Fluoresc. 1995, 5, 59–69. 10.1007/BF00718783. 24226612

[ref51] Krasnowska E. K.; Gratton E.; Parasassi T. Prodan as a Membrane Surface Fluorescence Probe: Partitioning between Water and Phospholipid Phases. Biophys. J. 1998, 74, 1984–1993. 10.1016/S0006-3495(98)77905-6. 9545057 PMC1299539

[ref52] Bagatolli L. A.; Parasassi T.; Fidelio G. D.; Gratton E. A Model for the Interaction of 6-Lauroyl-2-(N,N-Dimethylamino)Naphthalene with Lipid Environments: Implications for Spectral Properties. Photochem. Photobiol. 1999, 70, 557 10.1562/0031-8655(1999)070<0557:AMFTIO>2.3.CO;2. 10546552

[ref53] Parasassi T.; Gratton E.; Yu W. M.; Wilson P.; Levi M. Two-Photon Fluorescence Microscopy of Laurdan Generalized Polarization Domains in Model and Natural Membranes. Biophys. J. 1997, 72, 2413–2429. 10.1016/S0006-3495(97)78887-8. 9168019 PMC1184441

[ref54] Parasassi T.; De Stasio G.; d’Ubaldo A.; Gratton E. Phase Fluctuation in Phospholipid Membranes Revealed by Laurdan Fluorescence. Biophys. J. 1990, 57, 1179–1186. 10.1016/S0006-3495(90)82637-0. 2393703 PMC1280828

[ref55] Mély Y.; Duportail G.; Bagatolli L. A. Fluorescent Methods to Study Biological Membranes; Springer Berlin Heidelberg: Berlin, 2013.

[ref56] Gaus K.; Zech T.; Harder T. Visualizing Membrane Microdomains by Laurdan 2-Photon Microscopy. Mol. Membr. Biol. 2006, 23, 41–48. 10.1080/09687860500466857. 16611579

[ref57] Golfetto O.; Hinde E.; Gratton E. Laurdan Fluorescence Lifetime Discriminates Cholesterol Content from Changes in Fluidity in Living Cell Membranes. Biophys. J. 2013, 104, 1238–1247. 10.1016/j.bpj.2012.12.057. 23528083 PMC3602759

[ref58] Vequi-Suplicy C. C.; Lamy M. T.; Marquezin C. A. The New Fluorescent Membrane Probe Ahba: A Comparative Study with the Largely Used Laurdan. J. Fluoresc. 2013, 23, 479–486. 10.1007/s10895-013-1172-3. 23397490

[ref59] Ma Y.; Benda A.; Kwiatek J.; Owen D. M.; Gaus K. Time-Resolved Laurdan Fluorescence Reveals Insights into Membrane Viscosity and Hydration Levels. Biophys. J. 2018, 115, 1498–1508. 10.1016/j.bpj.2018.08.041. 30269886 PMC6257870

[ref60] Noutsi P.; Gratton E.; Chaieb S. Assessment of Membrane Fluidity Fluctuations during Cellular Development Reveals Time and Cell Type Specificity. PLoS One 2016, 11, e0158313 10.1371/journal.pone.0158313. 27362860 PMC4928918

[ref61] Jurkiewicz P.; Sýkora J.; Olzyńska A.; Humpolíčková J.; Hof M. Solvent Relaxation in Phospholipid Bilayers: Principles and Recent Applications. J. Fluoresc. 2005, 15, 883–894. 10.1007/s10895-005-0013-4. 16328702

[ref62] Sýkora J.; Kapusta P.; Fidler V.; Hof M. On What Time Scale Does Solvent Relaxation in Phospholipid Bilayers Happen?. Langmuir 2002, 18, 571–574. 10.1021/la011337x.

[ref63] McNeill C. R.; Abrusci A.; Hwang I.; Ruderer M. A.; Müller-Buschbaum P.; Greenham N. C. Photophysics and Photocurrent Generation in Polythiophene/Polyfluorene Copolymer Blends. Adv. Funct. Mater. 2009, 19, 3103–3111. 10.1002/adfm.200900801.

[ref64] Barrow D. A.; Lentz B. R. Membrane structural domains. Resolution limits using diphenylhexatriene fluorescence decay. Biophys. J. 1985, 48, 221–234. 10.1016/S0006-3495(85)83775-9. 4052559 PMC1329313

[ref65] Sinha M.; Mishra S.; Joshi P. G. Liquid-Ordered Microdomains in Lipid Rafts and Plasma Membrane of U-87 MG Cells: A Time-Resolved Fluorescence Study. Eur. Biophys. J. 2003, 32, 381–391. 10.1007/s00249-003-0281-3. 12851796

[ref66] Yao J.; Liu B.; Qin F. Rapid Temperature Jump by Infrared Diode Laser Irradiation for Patch-Clamp Studies. Biophys. J. 2009, 96, 3611–3619. 10.1016/j.bpj.2009.02.016. 19413966 PMC2711624

[ref67] Mosconi E.; Salvatori P.; Saba M. I.; Mattoni A.; Bellani S.; Bruni F.; Santiago Gonzalez B.; Antognazza M. R.; Brovelli S.; Lanzani G.; et al. Surface Polarization Drives Photoinduced Charge Separation at the P3HT/Water Interface. ACS Energy Lett. 2016, 1, 454–463. 10.1021/acsenergylett.6b00197.

[ref68] Wan A. M. D.; Schur R. M.; Ober C. K.; Fischbach C.; Gourdon D.; Malliaras G. G. Electrical Control of Protein Conformation. Adv. Mater. 2012, 24, 2501–2505. 10.1002/adma.201200436. 22489011

[ref69] Lodola F.; Martino N.; Tullii G.; Lanzani G.; Antognazza M. R. Conjugated Polymers Mediate Effective Activation of the Mammalian Ion Channel Transient Receptor Potential Vanilloid 1. Sci. Rep. 2017, 7, 8477 10.1038/s41598-017-08541-6. 28814817 PMC5559550

