# Supplementary material for: Shedding Light on Thermally Induced Optocapacitance at the Organic Biointerface
Source: J Phys Chem B. 2021 Sep 15;125(38):10748–58. doi: 10.1021/acs.jpcb.1c06054 (PMC8488932; doi:10.1021/acs.jpcb.1c06054)
Supplement: Supplementary file 1 — jp1c06054_si_001.pdf [file jp1c06054_si_001.pdf]

Supplementary Information

Shedding Light on Thermal Induced Optocapacitance at the Organic Bio-Interface

Gaia Bondelli[1, 2], Samim Sardar[2], Greta Chiaravalli[1,2], Vito Vurro[2], Giuseppe Maria Paternò[2*], Guglielmo Lanzani[1, 2*], Cosimo D'Andrea[1, 2*]

[1]Department of Physics, Politecnico di Milano, Milan, Italy

[2]Center for Nano Science and Technology @PoliMi, Istituto Italiano di Tecnologia, Milan, Italy

Corresponding Authors*:

cosimo.dandrea@polimi.it; guglielmo.lanzani@iit.it; giuseppe.paterno@iit.it

Supplementary Discussion

FINITE ELEMENT SIMULATION OF THERMAL DIFFUSION

The photoinduced temperature variation at the interface with the polymeric film was estimated using the COMSOL software. Heat diffusion has been modelled using standard heat diffusion theory and assuming that all the energy absorbed by the polymer (78% of the illumination, power density ≈35mW/mm^2) is transferred to the system as heat. Model parameters are listed in the following table:

Parameter	Electrolyte	P3HT:PCBM	Glass
**C_p [J kg^{-1} K^{-1}]**	$4.1813 \cdot 10^3$	$1.4 \cdot 10^3$	$0.84 \cdot 10^3$
**r [kg m^{-3}]**	1000	1100	2500
**k [W m^{-1} K^{-1}]**	0.6	0.2	1.0
**k [m^2 s^{-1}]**	$1.435 \cdot 10^{-7}$	$1.2987 \cdot 10^{-7}$	$4.7619 \cdot 10^{-7}$

Starting from room temperature (293.15 °K), the simulation reports a temperature increase equal to 15.77 °K, attained by the numerical solution in proximity of the solution/polymer interface at the center of the spotlight. The computed temperature variation as a function of time is reported in Supplementary Fig. 3.

DRIFT DIFFUSION SIMULATIONS

The PV measurement has been simulated with the use of a time dependent drift diffusion model for a bounded 1D domain, from x = 0 to x = L, being L the thickness of the device (~100 μm). The time domain goes from 0 to 5 s, with light shined from 0 to 3.5 s. The system of equation is formed by a Poisson equation:

$$\nabla_x \cdot D = q(p - n) \tag{1a}$$

$$D = \varepsilon E \tag{1b}$$

Being $\varepsilon = \varepsilon_r \varepsilon_0$ the dielectric constant of the medium [F/m], and ε_r the relative dielctric constant set equal to 3.5. In x = 0 we suppose E = 0 and we measure the photovoltage, being namely $\psi(x = 0, t)$, while in x = L we set the electric potential equal to the Open Circuit potential, namely 0.24 V vs Ag/AgCl sat. The continuity equations for holes (2a-2b) and electrons (3a-3b) are:

$$q\frac{\partial p}{\partial t} + \nabla_x \cdot J_p = q(k_{diss}X - \gamma np) \tag{2a}$$

$$J_p = -q\mu_p p\frac{\partial \psi}{\partial x} - qD_p\frac{\partial p}{\partial x} \tag{2b}$$

$$q\frac{\partial n}{\partial t} - \nabla_x \cdot J_n = q(k_{diss}X - \gamma np) \tag{3a}$$

$$J_n = -q\mu_n n\frac{\partial \psi}{\partial x} + qD_n\frac{\partial n}{\partial x} \tag{3b}$$

Being q the elementary charge [C], p and n the holes and electron number densities [m^{-3}]. k_{diss} is a lumped parameter describing the photon-free carriers efficiency of conversion, set equal to 1 in our case, while X describes the light intensity impinging onto the substrate in #photons/m^2s. γ is the Langevin bimolecular recombination coefficient, evaluated as min $(\mu_n, \mu_p)/(\varepsilon)$. The mobilities

μ_p and μ_n are related to the diffusion coefficients D_p,D_n with the Einstein-Smoluchowski relation and are set equal to $\mu_p = 1 \cdot 10^{-8}$, $\mu_n = 1 \cdot 10^{-7}$ $m^2\ V^{-1}s^{-1}$. In x = 0 J_p is set equal to zero, $J_n = 7 \cdot 10^{-25}\exp -\frac{t}{\tau}$, with $\tau = 1\ s$, representing a capacitive current entering the layer of ITO and necessary to describe the initial negative spike of the photopotential. In x = L, electrons exit the bulk as described with the Marcus-Gerischer theory in eq. (4) of the main text, while holes recombine as a function of the negative accumulated charge at the interface, namely:

$$J_p(L,t) = \ -k_p\sigma_s(t)p(L,t) \tag{4}$$

Where k_p is estimated by comparing the simulations with the experiments and $\sigma_s(t)$ is computed by solving a differential equation.

$$\frac{d\sigma_S}{dt} = J_n(L,t) \cdot nn + J_{p(L,t)} \cdot nn \tag{5}$$

Being *nn* the outward normal of the system.

SUPPLEMENTARY FIGURES

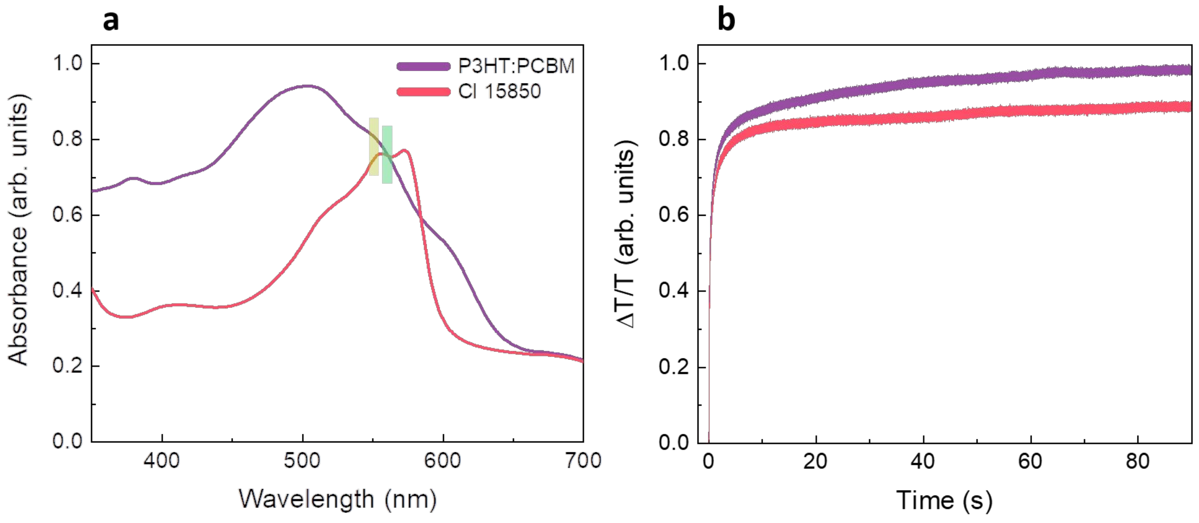

Figure S1: (**a**) Absorbance spectra of the two active layers used in the study, P3HT:PCBM and CI 15850. The green box represents the wavelength used to excite the two samples in TRPL experiments (λ = 561 nm), while the yellow box accounts for the wavelength used to excite the samples in calibrated pipette measurements (λ = 550 nm). (**b**) Photoinduced temperature variation in the two films, measured through calibrated pipette technique[3], reported as $\Delta T/T$. Temperature increase of the bath at the polymer interface is measured: a patch pipette is micromanipulated in close proximity (~ 2 μm) of the active material, with both the pipette and the bath filled with a KRH aqueous solution. Due to the temperature increase in the bath upon illumination of the active material, the resistance of the pipette decreases. To extract the actual temperature, the pipette resistance is pre-calibrated by measuring the variation in pipette current by controlling the bath temperature. The light source consisted of a LED with central wavelength at 550 nm and power density of 45 mW/mm^2.

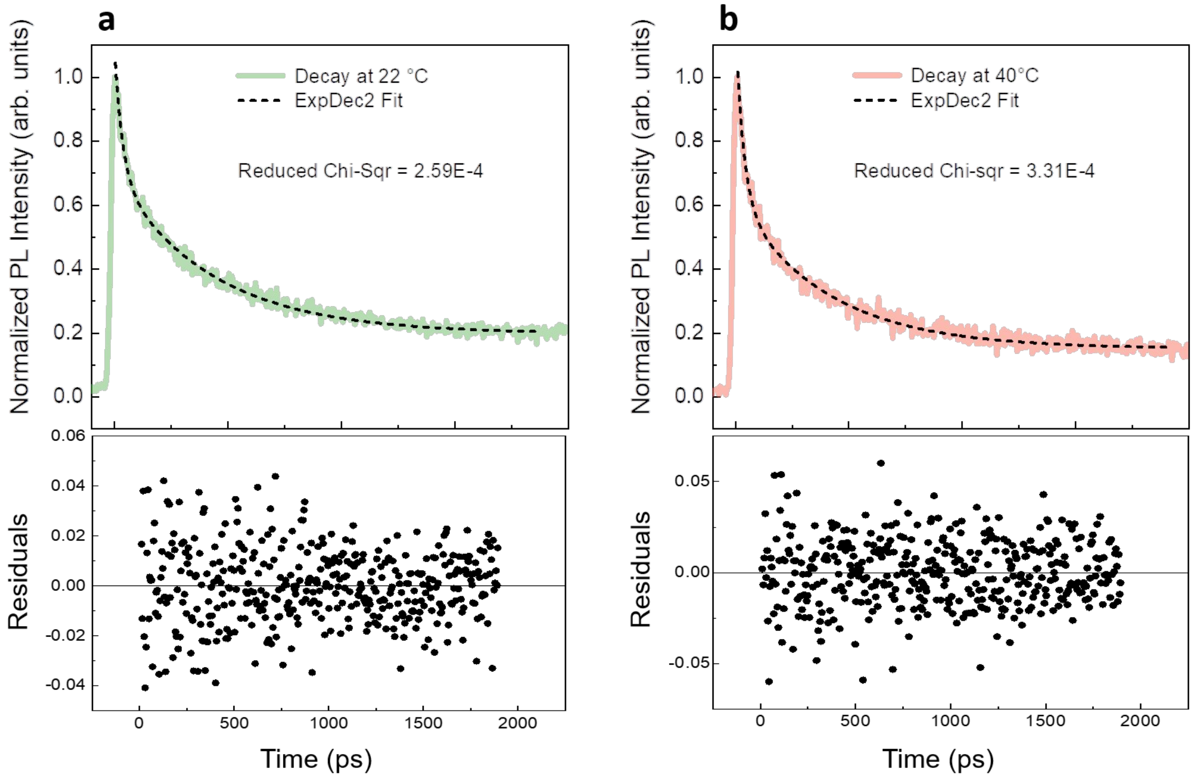

Figure S2: Bi-exponential decay fittings and residuals distributions of Laurdan PL lifetime in HEK-293 cells plated on glass substrates and measured at 22 °C (**a**) and 40 °C (**b**), obtained by Peltier plate heating.

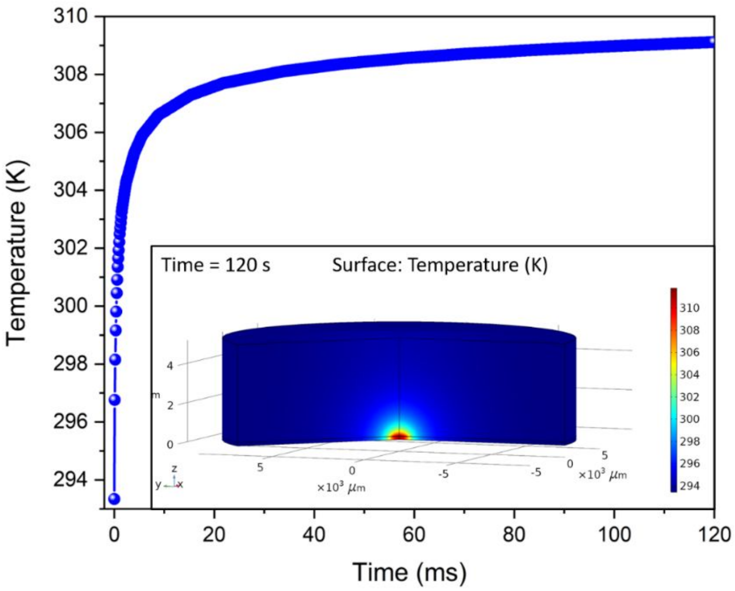

Figure S3: Temperature variation at the interface with the P3HT:PCBM film as a function of time, extracted from the finite element simulation (see above section). **Inset**: spatial distribution of the temperature profile of the system during illumination, simulated by COMSOL software.

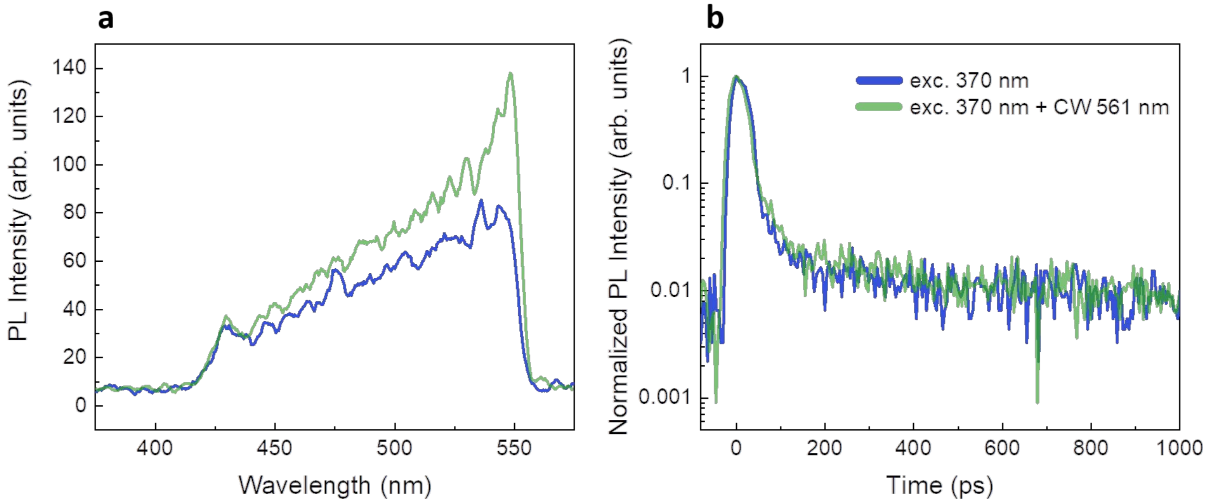

Figure S4: Control measurements on P3HT:PCBM film + aqueous buffer solution (no presence of cells), integrated in the full range -100 – 2000 ps (**a**) and 400-650 nm (**b**). PL contribution from the blend is present only in the first ~ 100 ps, which could be easily discarded. It has to be noted

that when measuring cells, the focal plane of the objective is different from that of the measurement on P3HT:PCBM film alone. As a consequence, the emission contribution from the blend film in actual measurements on cells is negligible.

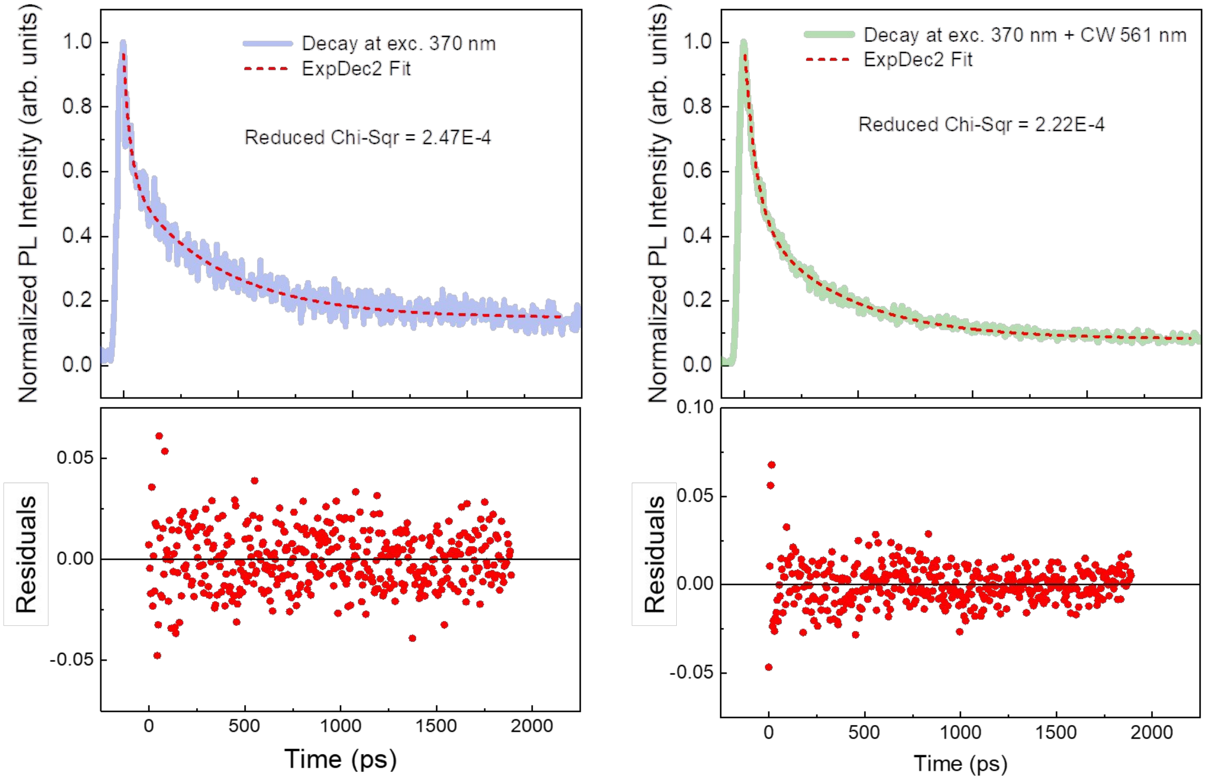

Figure S5: Bi-exponential decay fittings and residuals distributions of Laurdan PL lifetime in HEK-293 cells plated on P3HT:PCBM substrates upon excitation at 370 nm pulsed laser (**a**) and 370 nm + CW 561 nm lasers together (**b**).

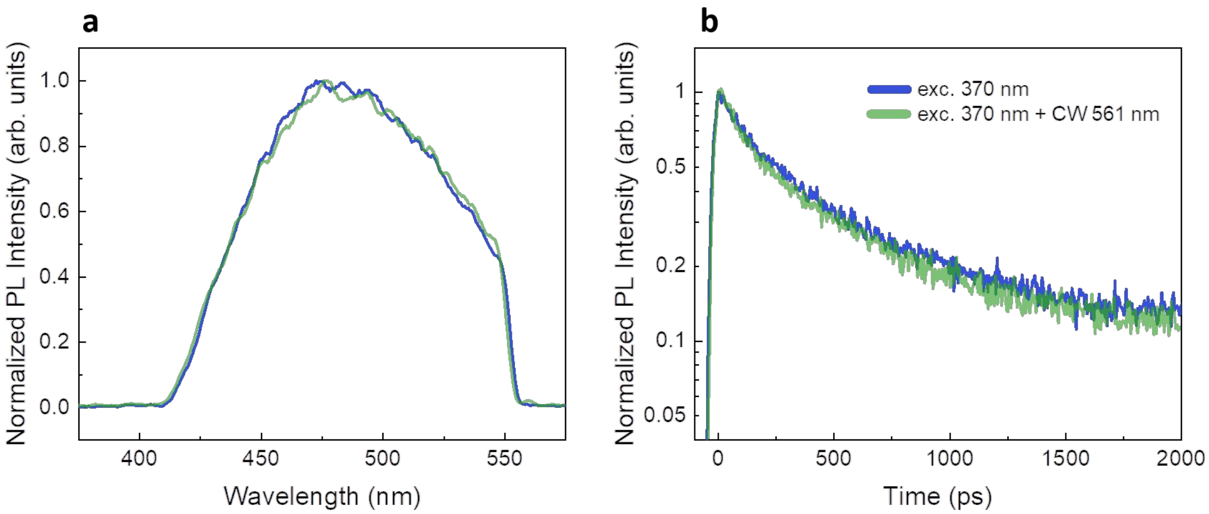

Figure S6: Control measurements on glass samples upon excitation at both 370 nm and CW 561 nm (power density = 46 mW/mm^2) lasers, to assess the implication of the P3HT:PCBM film in the observed changes in spectra shape and dynamics, showing no significant effects. Integration ranges are -100 – 2000 ps (**a**) and 440-650 nm (**b**).

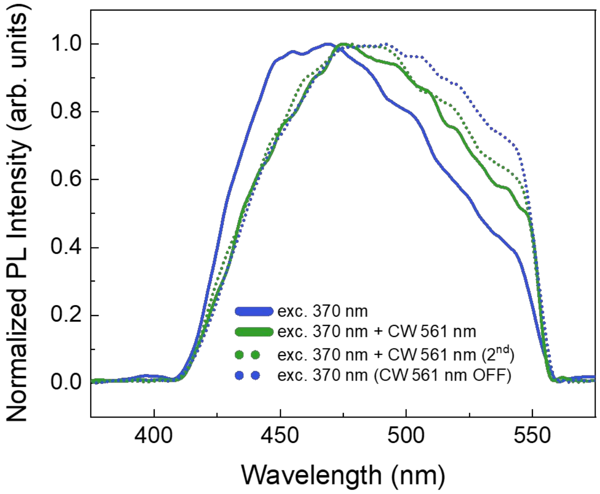

Figure S7: Control measurements on P3HT:PCBM samples, performed on the same cell spot. The Integration range is 100-2000 ps, to avoid polymeric film PL contribution. The blue and green continuous lines represent, respectively, a 1st measurement with excitation at 370 nm only and a subsequent measurement with 561 nm CW laser as well; the green dotted line represent a second measurement still performed with both lasers, immediately after the end of the previous one (about 120 seconds). Finally, the blue dotted curve accounts for a final measurement performed with exc. 370 nm only, immediately after the switching off the 561 nm CW laser. No reversibility was observed within the measured time window and no difference between the two measurements with 561 nm excitation. CW laser power is 46 mW/mm^2.

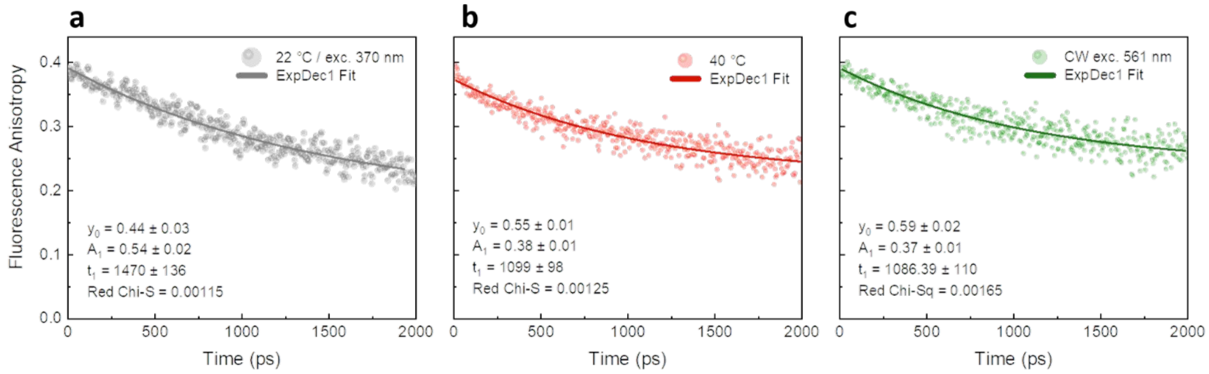

Figure S8: Decay of fluorescence anisotropy of TMA-DPH in HEK-293 cells at (**a**) 22 °C (exc. 370 nm only), (**b**) 40 °C, obtained through Peltier plate heating, and (**c**) P3HT:PCBM film upon excitation at 370 nm and CW 561 nm lasers. Dots represents the experimental data and solid lines represent the fitted curves. As the fluorescence characteristics of TMA-DPH are sensitive to its environment, it has been widely used to measure the order of lipid acyl chains in cell membranes and intact cells. For these experiments, samples were excited at 370 nm with vertically polarized light and vertical ($I(t)_{vv}$) and horizontal ($I(t)_{vh}$) polarized fluorescence intensities were measured, through the use of two polarizers placed in the optical paths. Rotational mobility of the probe was determined by measuring the time-resolved anisotropy, calculated as reported in (4) (Methods section). TMA-DPH is affected by considerable photo bleaching induced by excitation with UV light[1]. The quenching effects result from Förster-type fluorescence resonant auto-transfer[2]. For this reason, we summed together anisotropy data from several (n = 10) identical measurements, in order to reduce noise caused by the reduced probe signal.

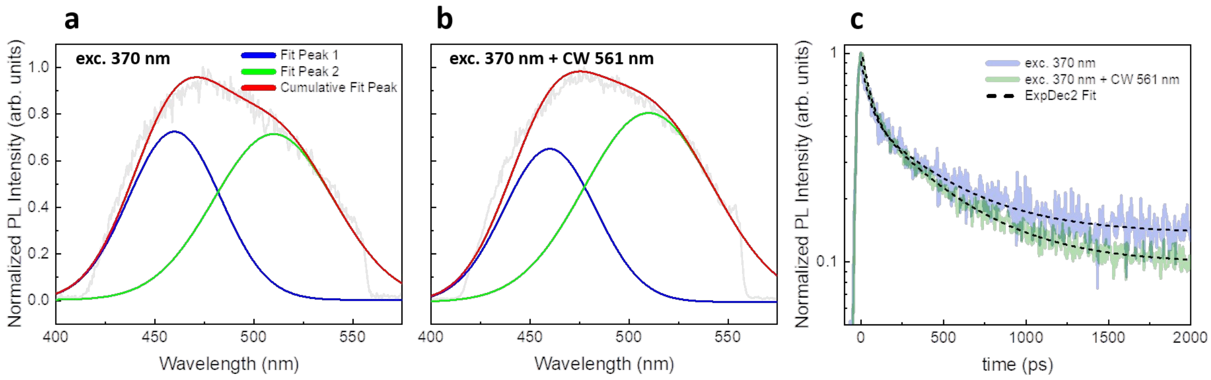

Figure S9: TRPL measurement on HEK-293 cells grown on CI 15850 films. Decomposition of the Laurdan emission spectra, recorded with excitation at 370 nm only (**a**) and 370 nm + CW 561 nm (46 mW/mm^2) (**b**), fitted with two Gaussian curves centered at 460 nm and 510 nm, used in the GP evaluation. Measurements were performed on the same cell spot. (**c**) Laurdan PL kinetics, integrated in the range 440-550nm, fitted to double exponential decay curves.

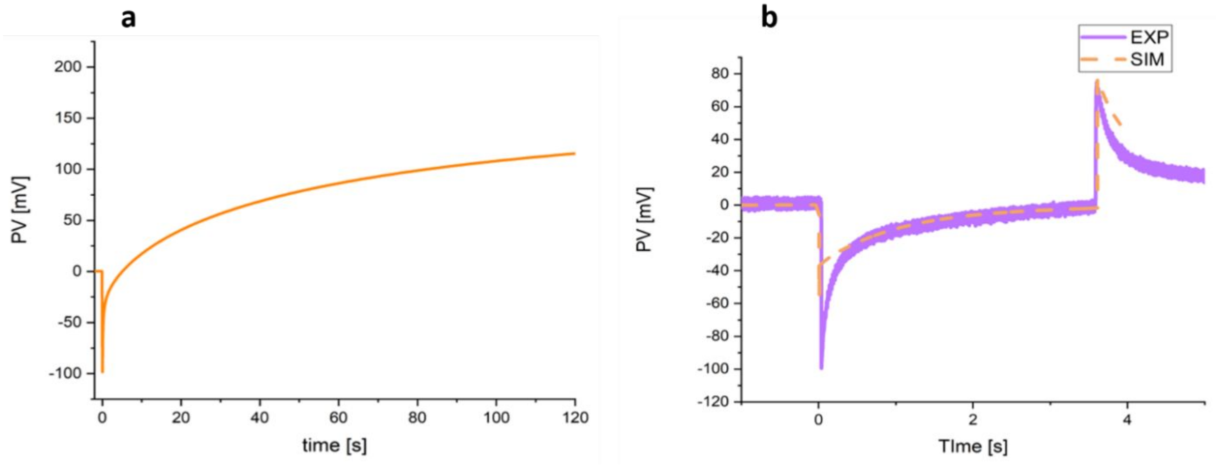

Figure S10: (a) Photovoltage measurement of ITO/P3HT:PCBM sample with 120 s of green LED light illumination starting from t=0. **(b)** ITO/P3HT:PCBM sample photovoltage measurement with 3.5 s illumination (purple curve) and photovoltage simulation with the Drift-Diffusion model described in the above section (orange dashed curve). From the fitting of the simulation with the experimental data, the value of k_t and the one of the holes recombination time constant at the interface, k_p, have been retrieved, and set equal to $k_t = 3 \cdot 10^{-29}$ m^4s^{-1} and $k_p = 7 \cdot 10^{-25}$ m^3s^{-1}, in order to estimate the accumulated charge after 120s of illumination.

The simulation provides an estimation of the accumulated charge at the interface after 120 s of illumination, $\sigma = 8 \cdot 10^{-2}$ Cm^{-2}. This value is coherent with the one retrieved from the experimental PV according to two different approaches. The first approach is based on the equation:

$$\sigma = \frac{C \times PV}{A} = 3.7 \cdot 10^{-2} Cm^{-2} \quad (6)$$

where $C = 3.1 \cdot 10^{-7}$ F is the estimated interface capacitance, PV the measured value and A the device area. The other approach considers two discs with surface charge σ, of opposite sign, separated by a distance δx. For distances x from the surface such as $x >> \delta x$, the electrostatic voltage is given by

$$\tilde{V}(x) = V(x) - V(x+\delta x) = -\frac{\partial V}{\partial x}\delta x \quad (7)$$

Thus:

$$\tilde{V}(x) = -\frac{\sigma \cdot \delta x}{2\varepsilon}\left(\frac{x}{\sqrt{R^2+x^2}} - 1\right) \quad (8)$$

Here *δx* is the effective separation of the negative and positive charge at the polymer-electrolyte interface. The negative charge will be distributed within the diffuse interface at the electrolyte, while the positive charge spreads in the polymer bulk. The polarized film generates a weak electric potential, assuming for R = 15 μm and a distance x = 0.2 mm the measured voltage depends on the separation *δx*. If we assume *δx=1 nm* we get V(200 μm) ≈ -30 mV.

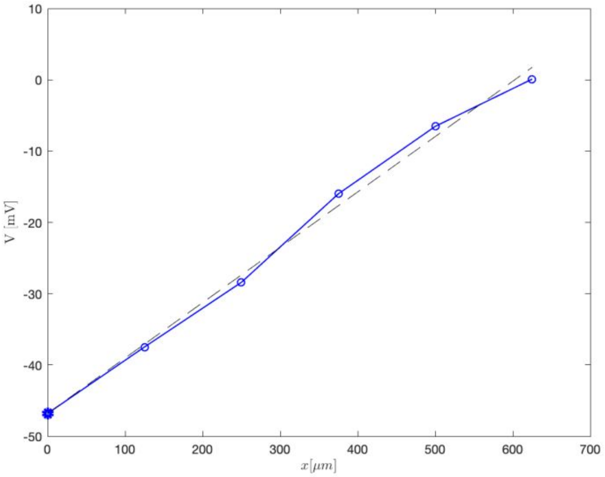

Figure S11: Zeta Surface Potential measurement of P3HT:PCBM in a water based solution with Silica nanoparticles. The blue empty dots show the measured values (shifted by the tracer potential as in eq. (3) of the main text) at 125, 250, 375, 500 and 625 μm; the full-blue dot in x = 0 shows the surface potential obtained with a linear extrapolation from the datasets (see the dashed black line). The data reported already show the measurements without the electrophoretic contribution of the Silica nanoparticles, computed in agreement with equation (3) of the main text.

SUPPLEMENTARY TABLES

Table S1: Relative area average values (n = 6) of the two Gaussian peaks, centred at 460 nm and 510 nm, obtained from the deconvolution of the experimentally obtained Laurdan emission spectra at 22 °C and 40 °C, with HEK-293 cells plated on glass substrates.

	**22 °C**	**40 °C**
**peak @ 460 nm**	49.04 ± 3	39.85 ± 3.5
**peak @ 510 nm**	33.35 ± 1.4	39.64 ± 2

Table S2: Relative area average values (n = 4) of the two Gaussian peaks, centred at 460 nm and 510 nm, obtained from the deconvolution of the experimentally obtained Laurdan emission spectra at 22 °C and 40 °C, with HEK-293 cells plated on P3HT:PCBM substrates.

	**22 °C**	**40 °C**
**peak @ 460 nm**	49.39 ± 1.1	43.11 ± 1.3
**peak @ 510 nm**	36.0 ± 3.4	39.53 ± 3.4

Table S3: Relative area average values (n = 6) of the two Gaussian peaks, centred at 460 nm and 510 nm, obtained from the deconvolution of the experimentally obtained Laurdan emission spectra in P3HT:PCBM samples upon excitation with 370 nm laser and 370 nm + CW 561 nm lasers.

	**exc. 370 nm**	**exc. 370 nm + CW 561**
**peak @ 460 nm**	50.36 ± 2.3	39.03 ± 1.5
**peak @ 510 nm**	32.70 ± 4.3	51.32 ± 1.9

Table S4: Averaged results (n = 6) of the double exponential fitting of Laurdan PL decay curves obtained exciting P3HT:PCBM samples with different CW 561 nm laser powers.

Excitation	y_0	A_1	τ_1 (ps)	A_2	τ_2 (ps)	$\Delta\tau_2$ (%)
**370 nm**	0.14 ± 0.03	0.35 ± 0.05	33 ± 16	0.49 ± 0.06	423 ± 27	/
**370 nm + CW 561 nm (4.5 mW/mm²)**	0.12 ± 0.03	0.42 ± 0.15	31 ± 12	0.47 ± 0.11	409 ± 27	-3.31
**370 nm + CW 561 nm (8.6 mW/mm²)**	0.09 ± 0.02	0.63 ± 0.20	30 ± 5	0.33 ± 0.13	372 ± 21	-12.04
**370 nm + CW 561 nm (17 mW/mm²)**	0.09 ± 0.04	0.51 ± 0.08	32 ± 3	0.42 ± 0.10	353 ± 19	-16.59
**370 nm + CW 561 nm (29 mW/mm²)**	0.09 ± 0.02	0.37 ± 0.02	35 ± 15	0.46 ± 0.03	341 ± 35	-19.43
**370 nm + CW 561 nm (46 mW/mm²)**	0.08 ± 0.02	0.49 ± 0.10	33 ± 8	0.47 ± 0.05	332 ± 45	-21.69

Table S5: Relative area average values (n = 6) of the two Gaussian peaks, centred at 460 nm and 510 nm, obtained from the deconvolution of the experimentally obtained Laurdan emission spectra in CI 15850 samples upon excitation with 370 nm laser and 370 nm + CW 561 nm lasers.

	**exc. 370 nm**	**exc. 370 nm + CW 561**
**peak @ 460 nm**	58.15 ± 2.3	48.04 ± 3.5
**peak @ 510 nm**	42.06 ± 2.6	46.63 ± 4.2

SUPPLEMENTARY REFERENCES

1. Duportail, G. & Weinreb, A. Photochemical changes of fluorescent probes in membranes and their effect on the observed fluorescence anisotropy values. *Biochim. Biophys. Acta* **736**, 171–177 (1983).

2. Illinger, D. *et al.* A comparison of the fluorescence properties of TMA-DPH as a probe for plasma membrane and for endocytic membrane. *BBA - Biomembr.* **1239**, 58–66 (1995).

3. Yao, J., Liu, B. & Qin, F. Rapid temperature jump by infrared diode laser irradiation for patch-clamp studies. *Biophys. J.* **96**, 3611–3619 (2009).
